# Maf1‐dependent transcriptional regulation of tRNAs prevents genomic instability and is associated with extended lifespan

**DOI:** 10.1111/acel.13068

**Published:** 2019-12-12

**Authors:** Mihir Shetty, Chiaki Noguchi, Sydney Wilson, Esteban Martinez, Kazuhiro Shiozaki, Christian Sell, Joshua Chang Mell, Eishi Noguchi

**Affiliations:** ^1^ Department of Biochemistry and Molecular Biology Drexel University College of Medicine Philadelphia PA USA; ^2^ Division of Biological Science Nara Institute of Science and Technology Ikoma Japan; ^3^ Department of Microbiology and Molecular Genetics University of California Davis CA USA; ^4^ Department of Pathology Drexel University College of Medicine Philadelphia PA USA; ^5^ Department of Microbiology and Immunology Centers for Genomics Sciences Drexel University College of Medicine Philadelphia PA USA; ^6^ Present address: Fox Chase Cancer Center Philadelphia PA USA

**Keywords:** aging, DNA damage, DNA repair, lifespan, Maf1, RNA polymerase III, transcription, tRNA

## Abstract

Maf1 is the master repressor of RNA polymerase III responsible for transcription of tRNAs and 5S rRNAs. Maf1 is negatively regulated via phosphorylation by the mTOR pathway, which governs protein synthesis, growth control, and lifespan regulation in response to nutrient availability. Inhibiting the mTOR pathway extends lifespan in various organisms. However, the downstream effectors for the regulation of cell homeostasis that are critical to lifespan extension remain elusive. Here we show that fission yeast Maf1 is required for lifespan extension. Maf1’s function in tRNA repression is inhibited by mTOR‐dependent phosphorylation, whereas Maf1 is activated via dephosphorylation by protein phosphatase complexes, PP4 and PP2A. Mutational analysis reveals that Maf1 phosphorylation status influences lifespan, which is correlated with elevated tRNA and protein synthesis levels in *maf1*∆ cells. However, mTOR downregulation, which negates protein synthesis, fails to rescue the short lifespan of *maf1*∆ cells, suggesting that elevated protein synthesis is not a cause of lifespan shortening in *maf1*∆ cells. Interestingly, *maf1*∆ cells accumulate DNA damage represented by formation of Rad52 DNA damage foci and Rad52 recruitment at tRNA genes. Loss of the Rad52 DNA repair protein further exacerbates the shortened lifespan of *maf1*∆ cells. Strikingly, PP4 deletion alleviates DNA damage and rescues the short lifespan of *maf1*∆ cells even though tRNA synthesis is increased in this condition, suggesting that elevated DNA damage is the major cause of lifespan shortening in *maf1*∆ cells. We propose that Maf1‐dependent inhibition of tRNA synthesis controls fission yeast lifespan by preventing genomic instability that arises at tRNA genes.

## Graphical abstract

In response to nutrient availability, mTOR inactivates Maf1 via phosphorylation. This results in hyperactivation of Pol III‐mediated transcription, leading to DNA damage due to collisions between the replication and transcription machineries. Such DNA damage may shorten lifespan. mTOR‐mediated activation of cellular processes and increased energy expenditure also negatively affect lifespan.

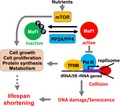

## 1 INTRODUCTION

Fundamental cellular mechanisms such as nutrient sensing, DNA damage response pathways, and cell cycle regulation influence the aging process. Studies have shown that the nutrient sensory kinase, mTOR (TOR in yeast), regulates lifespan in response to nutrient availability. The mTOR kinase forms two distinct protein complexes: TORC1 and TORC2. TORC1, which is inhibited by rapamycin, regulates cell growth, proliferation, and metabolism. It is well established that TORC1 promotes protein translation via phosphorylation of ribosomal protein S6 kinase and the eIF4E‐binding protein (BP; Zoncu, Efeyan, & Sabatini, 2011). The TORC2 branch is less studied; however, TORC2 also plays important roles in metabolism, cell survival, and proliferation (Zoncu et al., 2011). Although the involvement of the TORC1 pathway in lifespan regulation is conserved among many species (i.e., TORC1 inhibition extends lifespan), it is still unclear how this pathway affects multiple downstream stress and damage response mechanisms.

One of the known TORC1 targets is the Maf1 protein, which represses RNA polymerase III (Pol III)‐mediated transcription including tRNAs and 5S rRNA in response to a variety of stresses, including nutrient deprivation (Michels, 2011). Maf1 is regulated via phosphorylation by the TORC1 pathway and dephosphorylation by PP2A and PP4 in response to nutrient availability. When the TORC1 pathway is inhibited, Maf1 becomes dephosphorylated, thus activated. In this condition, Maf1 exerts its ability to repress Pol III‐mediated transcription (Zhang, Li, Wang, & Steven Zheng, 2018). Because calorie or dietary restriction inhibits the TORC1 pathway to extend lifespan (Kaeberlein & Kennedy, 2011), one can speculate that Maf1‐mediated Pol III inhibition could extend lifespan. Consistent with this, a recent study reported that Pol III inhibition extends lifespan in *Saccharomyces cerevisiae*, *Caenorhabditis elegans*, and *Drosophila* (Filer et al., 2017). Interestingly, limiting Pol III activity in intestinal cells is sufficient to extend lifespan in nematode worms and fruit flies, and Maf1‐overexpression reduces tRNA transcripts in fruit fly guts, which accompanies extended lifespan. This study also suggested that reduced protein synthesis via Pol III inhibition could lead to lifespan extension (Filer et al., 2017); however, how and why Maf1‐mediated Pol III inhibition influences lifespan is still elusive.

In budding yeast, studies showed that *maf1*∆ cells, which have elevated levels of Pol III‐mediated transcripts, have a shorter chronological lifespan (CLS) than wild‐type cells under both high‐calorie (2% glucose) and calorie‐restricted conditions (Cai & Wei, 2015, 2016), suggesting that Maf1‐mediated Pol III inhibition prevents lifespan shortening. This is consistent with the lifespan extension by Pol III inhibition in budding yeast, nematodes, and fruit flies (Filer et al., 2017). However, *mafr‐1* (the Maf1 ortholog) inhibition extends lifespan in *C. elegans*, even though Pol III activity is elevated in this organism. This is attributed to elevated oxidative stress response, mitochondrial unfolded protein response, and autophagy, which altogether seem to attenuate negative effects due to *mafr‐1* inhibition (Cai & Wei, 2016). In mice, Maf1 knockout alters insulin signaling and prevents diet‐induced obesity. Maf1^−/−^ mice also have elevated autophagy, leading to a lifespan extension when they are fed with the standard chow diet. These health benefits appear to be due to the increased turnover of tRNAs and lipids (Bonhoure et al., 2015). However, in worm and mammalian cells, Maf1 knockout results in lipogenic gene expression and lipid accumulation (Khanna, Johnson, & Curran, 2014; Palian et al., 2014), while in *Drosophila*, Maf1 inhibition stimulates insulin signaling, leading to increased growth and body mass (Rideout, Marshall, & Grewal, 2012). These discrepancies are probably due to the differences in diets/nutrients, growth conditions, and organisms. Thus, the precise role of Maf1 in lifespan and growth control remains elusive.

In the present study, we investigated the role of Maf1 in lifespan regulation using the fission yeast *Schizosaccharomyces pombe*, a popular model organism to study biological processes (Hoffman, Wood, & Fantes, 2015). We show that *maf1*∆ cells fail to both repress tRNA synthesis and to extend lifespan. We also show that Maf1 phosphorylation status dictates tRNA levels and lifespan. Strikingly, the short lifespan of *maf1*∆ cells is not rescued by inhibition of the TORC1 pathway. Unexpectedly, the lifespan shortening in *maf1*∆ cells does not appear to be due to elevated levels of protein synthesis. Rather, our results suggest that transcription‐mediated DNA damage accumulated in *maf1*∆ cells is the major cause of lifespan shortening. We propose that Maf1‐dependent transcriptional regulation of tRNA genes prevents genomic instability and extends lifespan in *S. pombe*. Given that tRNA regulation and the aging processes are largely conserved among eukaryotic species, our studies provide important information to shape future studies in mammalian models.

## 2 RESULTS

### 2.1 Maf1 inhibits tRNA transcription under calorie‐restricted conditions in *S. pombe*

**Figure 1:**
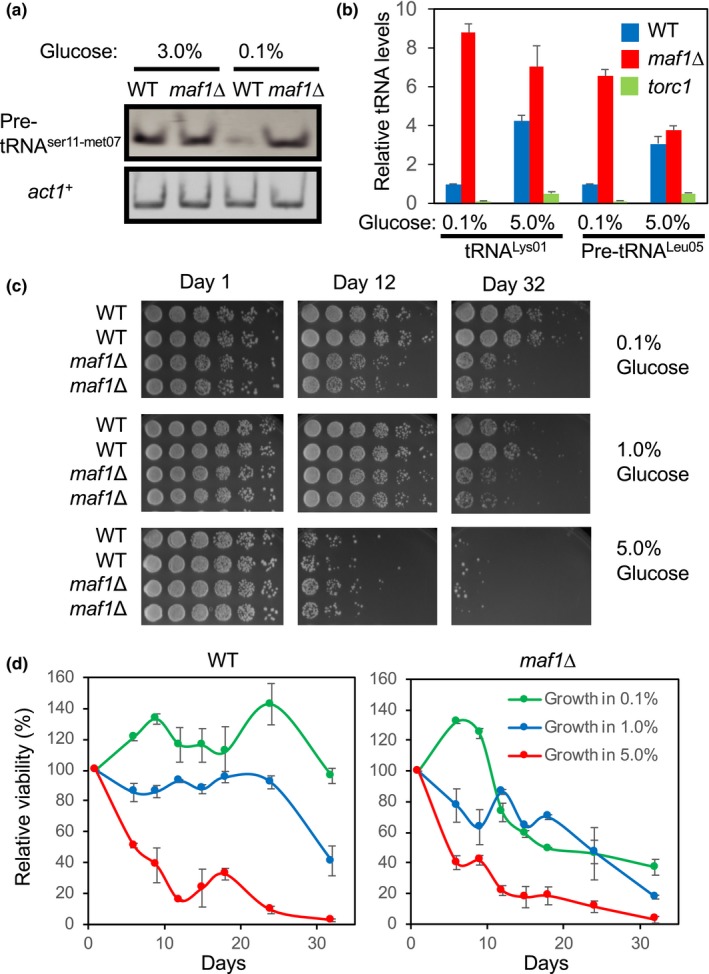
Loss of Maf1 shortens chronological lifespan. (a) WT and *maf1*Δ cells were cultured in YES medium containing 3% or 0.1% glucose overnight at 30°C. Levels of the pre‐tRNA^ser11‐met07^ and *act1* ^+^ genes were examined by RT–PCR from total RNA preparation. tRNA samples were run on acrylamide gels and stained by SYBR Green. (b) WT and *maf1*Δ cells were cultured overnight in YES medium with 0.1% or 5.0% glucose. Expressions of the pre‐tRNA^Leu05^ and tRNA^Lys01^ were examined by RT–PCR. The histograms show the relative tRNA expression levels normalized to *act1^+^* expression. Data are expressed as the mean of three independent experiments. Error bars represent the standard error of the mean (*SEM*). (c) WT and *maf1*Δ cells were first cultured at 30°C in YES liquid medium with different percentages of glucose (0.1%, 1%, and 5%) for the indicated days. Fivefold serial dilutions of the cells were then plated on YES agar medium containing 3% glucose, incubated for 3 days at 30°C, and photographed. The complete presentation of the results is shown in Figure S2. (d) Quantification of the lifespan assays shown in Figure S2 was performed by using NIH Image J. The average growth intensity of each strain on each day was obtained from colonies derived from six dilutions. To calculate the average deviation (error bar), two strains for each genotype were tested, and their growth was quantified

Maf1 inhibits tRNA transcription in *S. cerevisiae*, *Drosophila*, *C. elegans*, mice, and human cells under various stress conditions including calorie restriction (Khanna, Pradhan, & Curran, 2015). A recent report also showed that rapamycin decreases tRNA transcription in a Maf1‐dependent manner in *S. pombe* (Arimbasseri et al., 2015). Therefore, we first determined whether Maf1 is responsible for tRNA repression under calorie‐restricted conditions in *S. pombe*. For this purpose, we examined tRNA transcript (precursor tRNA or pre‐tRNA) levels as a readout of Pol III activity in wild‐type and *maf1*∆ cells under glucose‐rich (3.0% glucose, high calorie) or ‐poor conditions (0.1% glucose, calorie‐restricted). Wild‐type cells repressed dimeric pre‐tRNA^ser11–met07^ levels when glucose is limited (calorie‐restricted), whereas *maf1*∆ cells failed to inhibit tRNA transcription in the calorie‐restricted condition (Figure 1a). There was no significant difference between wild‐type and *maf1*∆ in the expression of the *act1* ^+^ gene, which is transcribed by Pol II. We also measured levels of pre‐tRNA^leu05^ and tRNA^lys01^ and found that *maf1*∆ cells have dramatically increased levels of tRNA transcription compared with wild‐type cells especially when glucose is limited (0.1% glucose; Figure 1b). Thus, Maf1 represses tRNA transcription under calorie‐restricted conditions.

### 2.2 Maf1 is required for lifespan extension

TORC1 plays a critical role in lifespan regulation under calorie‐restricted conditions, and calorie restriction extends lifespan in various organisms including *S. pombe* (Chen & Runge, 2009). Therefore, we hypothesized that Maf1 is involved in lifespan regulation in *S. pombe*. We first determined the effect of glucose on *S. pombe* growth rate. As reported in previous studies (Roux et al., 2009), irrespective of glucose content in the medium, wild‐type *S. pombe* cells approached stationary phase after approximately 2 days (Figure S1a). However, cells approached stationary phase at higher cell densities in high‐glucose medium compared with low‐glucose medium (Figure S1a). In addition, there was no significant difference in growth phenotypes between wild‐type and *maf1*∆ cells (Figure S1b). We then determined the effect of *maf1* deletion on lifespan. For this purpose, we used CLS to evaluate fission yeast lifespan. It is important to note that budding yeast, another important model organism, is known to age replicatively and chronologically. Replicative lifespan (RLS) is defined as the number of times a single cell divides prior to senescence, while CLS is defined as the length of time that a cell remains viable in G_0_ phase or nondividing phase (Carmona‐Gutierrez & Buttner, 2014). However, a complete pedigree analysis demonstrated that fission yeast does not age replicatively unless stressed (Coelho et al., 2013).

Consistent with previous studies (Chen & Runge, 2009; Roux et al., 2009), wild‐type *S. pombe* cells displayed an extended lifespan as the glucose concentration was reduced in the medium (Figures 1c, d and S2). However, this lifespan extension effect was largely diminished in *maf1*∆ cells (Figures 1c, d and S2). Furthermore, the short lifespan of *maf1*∆ cells was rescued by introducing the *maf1* gene from a plasmid, which was integrated into the *S. pombe* genome (Figure S1c). Therefore, consistent with the previous finding in *S. cerevisiae* (Cai & Wei, 2016), our data indicate that *S. pombe* Maf1 is required for the extension of CLS particularly under lower glucose conditions.

### 2.3 Maf1 phosphorylation is regulated by TORC1 and PP2A/PP4 phosphatases in response to glucose concentration changes in *S. pombe*

**Figure 2:**
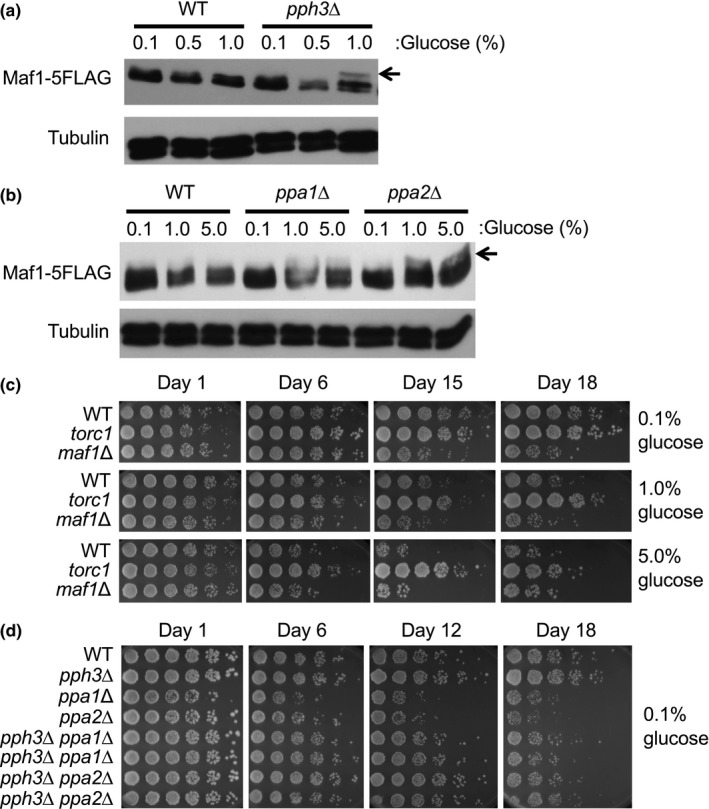
Maf1 is regulated by PP2A/PP4 phosphatases and the TORC1 kinase. (a, b) Cells of the indicated genotypes were engineered to express Maf1–5FLAG and grown at 30°C in YES liquid medium supplemented with 0.1%, 1%, or 5% glucose. Protein extracts were prepared, and Western blotting of Maf1‐5FLAG was performed. Tubulin was used as a loading control. Representative images of repeat experiments are shown. (c) WT, *torc1*, and *maf1*Δ cells were cultured in YES medium with different percentages of glucose (0.1%, 1%, and 5%) at 25°C for the indicated days. Fivefold serial dilutions of the cells were then plated on YES agar medium containing 3% glucose for lifespan assays. (d) Lifespan assay was performed with cells of the indicated genotypes. Cells were cultured in YES liquid medium supplemented with 0.1% glucose at 30°C for the indicated days before plating on YES agar medium as described above. Representative images of repeat experiments are shown

Earlier studies have shown that TORC1 is involved in Maf1 phosphorylation (thus inactivation). Under nutrient‐deprived conditions or in response to stress, PP2A and PP4 phosphatase complexes promote Maf1 dephosphorylation (thus activation; Zhang et al., 2018). To investigate Maf1 phosphorylation in *S. pombe*, Maf1 was fused to five tandem copies of the FLAG epitope (Maf1–5FLAG) and expressed at endogenous levels from its own promoter at the *maf1* locus. The *maf1–5FLAG* gene rescued the short lifespan of *maf1*∆ cells (Figure S1c), indicating that the Maf1–5FLAG protein is functional. We examined the phosphorylation status of Maf1 under varying concentrations of glucose and found that slower‐migrating species of Maf1 increased as the concentration of glucose elevated. This effect was particularly obvious in the absence of Pph3, the catalytic subunit of the PP4 phosphatase (Figure 2a). To determine whether the slower‐migrating Maf1 is due to phosphorylation, Maf1–5FLAG was immunoprecipitated. Addition of λ phosphatase converted the slower‐migrating band into the faster‐migrating band (Figure S3a). This effect was eliminated in the presence of phosphatase inhibitors, indicating that the slower‐migrating species represents Maf1 phosphorylation (Figure S3a). We found that 1% glucose was sufficient to induce slower‐migrating Maf1 species in the absence of Pph3 (Figure 2a). We also observed increased Maf1 phosphorylation in the absence of PP2A catalytic subunits, Ppa1 or Ppa2 (Figure 2b). We then generated *pph3*∆ *ppa1*∆ and *pph3*∆ *ppa2*∆ double mutants and examined Maf1 phosphorylation; there was no further increase in Maf1 phosphorylation in these double‐mutant cells when compared to *pph3*∆ single mutants (Figure S3b). These results indicate that Maf1 is hyperphosphorylated at high‐calorie conditions, while it undergoes PP4‐ and PP2A‐dependent dephosphorylation under calorie‐restricted conditions in *S. pombe*.

We then investigated the role of TORC1 in Maf1 phosphorylation. A previous report used the *tor2.51* allele and showed that Maf1 is phosphorylated in a TORC1‐dependent manner (Du, Halova, Kirkham, Atkin, & Petersen, 2012). In this study, we used the *tor2‐L2048S* allele (*torc1*), which contains a point mutation in *S. pombe* Tor2, the catalytic subunit of TORC1 (Hayashi et al., 2007), to downregulate TORC1. When grown in the presence of 3% glucose, Maf1 phosphorylation, which was elevated in *pph3*∆ cells, was diminished in *torc1 pph3*∆ double mutants (Figure S3c). Therefore, consistent with the previous report, TORC1 is involved in the phosphorylation of Maf1 under high‐calorie conditions.

### 2.4 Role of kinases and phosphatases that modulate Maf1 in lifespan regulation

Because Maf1 phosphorylation affects its activity as an inhibitor of Pol III‐mediated transcription (Michels, 2011), and Pol III regulates lifespan downstream of TORC1 (Filer et al., 2017), we hypothesized that *S. pombe* lifespan correlates with Maf1 phosphorylation status. Consistent with this hypothesis, a previous study demonstrated a positive effect of rapamycin on *S. pombe* lifespan extension (Rallis, Codlin, & Bahler, 2013). Consequently, we examined the lifespan of TORC1 mutant. *torc1* mutant cells displayed a longer lifespan than wild‐type cells especially in the presence of 1% and 5% glucose (Figures 2c and S2). This result is consistent with the effect of TORC1 inhibition on lifespan regulation in other organisms.

Next, we examined the lifespan of phosphatase mutants including *pph3*∆ (PP4), *ppa1*∆ (PP2A), and *ppa2*∆ (PP2A) cells. Although *pph3*∆ cells did not show lifespan shortening when grown in the presence of 0.1% glucose, *ppa1*∆ and *ppa2*∆ cells were considerably short‐lived than wild‐type cells (Figure 2d). We also attempted to examine the lifespan of *ppa1*∆ *ppa2*∆ double‐mutant cells. However, *ppa1*∆ was synthetically lethal with *ppa2*∆ (data not shown). Nevertheless, these results indicate that PP2A plays a role in lifespan extension. Interestingly, *pph3*∆ cells consistently displayed a slightly longer lifespan than wild‐type cells (Figure 2d). We also generated *pph3*Δ *ppa1*Δ and *pph3*Δ *ppa2*Δ double mutants and tested their lifespan. *pph3*∆ partially rescued the short lifespan of *ppa1*∆ and *ppa2*∆ cells (Figure 2d), suggesting the role of Pph3 in lifespan shortening. Possible mechanistic insights from this finding are described later in further details.

### 2.5 Identification of Maf1 phosphorylation sites

**Figure 3:**
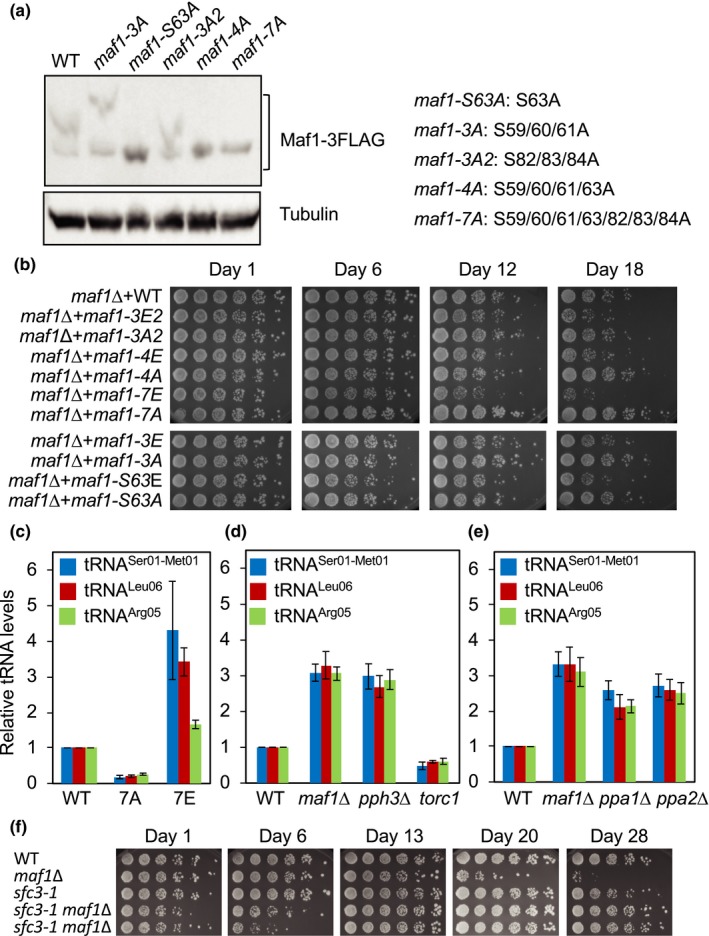
Maf1 phosphorylation dictates *Schizosaccharomyces pombe* lifespan and tRNA levels. (a) *maf1*Δ cells expressing Maf1–FLAG with the indicated mutations were cultured in YES medium with 3% glucose at 30°C. Equal amounts of protein were subjected to Western blotting and probed by the anti‐FLAG antibody. The mutations introduced in each Maf1 mutant are also indicated. (b) *maf1*Δ cells expressing Maf1–FLAG with the indicated mutations were cultured in YES liquid medium supplemented with 0.1% glucose at 30°C for the indicated days. For the lifespan assay, fivefold serial dilutions of cells were then plated on YES agar medium containing 3% glucose to evaluate viability. YES agar plates were incubated for 3 days at 30°C and photographed. (c) *maf1*Δ cells expressing Maf1–FLAG, Maf1‐7A–FLAG, and Maf1‐7E–FLAG were cultured in YES liquid medium supplemented with 0.1% glucose at 30°C. Expressions of the pretRNA^ser01‐met01^, pre‐tRNA^leu06^, and pre‐tRNA^arg05^ were examined. tRNA expression was normalized to *act1* ^+^ gene expression. Data are expressed as the mean of three independent experiments. Error bars represent standard error of the mean (*SEM*). (d, e) Cells of the indicated genotypes were cultured in YES liquid medium supplemented with 0.1% glucose at 30°C. tRNA expression analyses are performed as described in (b). (f) Lifespan assay of cells with the indicated genotypes was performed as described in (b). The complete presentation of the results is shown in Figure S5a

The above findings suggest that Maf1 phosphorylation correlates with *S. pombe* lifespan under calorie‐restricted conditions. Maf1 is evolutionarily conserved from yeast to humans, and several phosphorylation sites have been identified between domains A and B of Maf1 in humans and *S. cerevisiae* (Zhang et al., 2018; Figure S4a). In order to identify phosphorylation sites in *S. pombe* Maf1, we performed ClustalW multiple sequence alignment of Maf1 proteins from humans, *S. cerevisiae*, and *S. pombe* (Figure S4b). Based on the amino acid sequence similarities, we mutated serine residues (S) at 59th, 60th, 61st, 63rd, 82nd, 83rd, and 84th residues to alanine (A) in combination (Figures 3a and S4a). These mutated versions of the *maf1* gene were integrated into the *leu1* locus of *maf1*∆ cells and expressed as FLAG‐tagged proteins from the *maf1* promoter, in order to express Maf1 at its endogenous level. Accordingly, we generated *maf1‐S63A*, *maf1–S59/60/61A* (*maf1‐3A*), *maf1–S59/60/61/63A* (*maf1–4A*), *maf1–S82/83/84A* (*maf1–3A2*), and *maf1–S59/60/61/63/82/83/84A* (*maf1–7A*; Figure 3a). These mutations had no significant effect in the steady‐state level of Maf1 (Figure S3d). To increase the resolution of phosphorylation‐mediated mobility shift due to multiple phosphorylation sites, we utilized Phos‐tag (Kinoshita, Kinoshita‐Kikuta, Takiyama, & Koike, 2006) to analyze Maf1 phosphorylation. Interestingly, the single amino acid change (*maf1‐S63A*) eliminated the slow‐migrating form of Maf1 (Figure 3a). *maf1–4A* and *maf1–7A* mutants also include the S63A mutation and lost the slow‐migrating Maf1 band (Figure 3a). In contrast, the *maf1‐3A* and *maf1‐3A2* mutants, that do not contain the S63A mutation, still displayed the slow‐migrating Maf1 species (Figure 3a). We noticed that Maf1‐3A had a greater phosphorylation shift than wild‐type Maf1. It is possible that the *maf1‐3A* mutation causes conformational changes to allow additional phosphorylation of Maf1 although this is not the focus of this study. Nevertheless, our results are consistent with the notion that that S63A is the primary phosphorylation site of Maf1.

### 2.6 Phosphorylation status of Maf1 affects *S. pombe* lifespan

Next, we determined the role of Maf1 phosphorylation in *S. pombe* lifespan extension. Strikingly, *maf1–7A* displayed an extended lifespan compared with the wild‐type control (Figure 3b), indicating that Maf1 dephosphorylation is involved in lifespan extension. Although *maf1–S63A* and *maf1–4A* mutations also eliminated the mobility shift of Maf1 (Figure 3a), cells carrying these mutations failed to show a significant lifespan extension. Therefore, our findings suggest that, in addition to S63, phosphorylation at other sites also contribute to Maf1‐dependent lifespan regulation.

In order to mimic the phosphorylated state of Maf1, the phosphorylation sites were also changed from serine residues (S) to glutamic acids (E). Accordingly, *maf1–S63E*, *maf1–3E*, *maf1–4E*, *maf1‐3E2*, and *maf1–7E* mutants were generated. These mutants showed similar Maf1 levels compared to wild‐type cells (Figure S3d). Interestingly, there were electrophoretic mobility differences among the mutants in SDS‐PAGE analyses probably due to the conformational changes caused by S to E mutations (Figure S3d). Importantly, all these mutants were short‐lived compared with wild‐type cells. The strongest lifespan‐shortening effect was observed with the *maf1–7E* mutant (Figure 3b). These results indicate that Maf1 is phosphorylated at multiple serine residues and that Maf1 phosphorylation affects *S. pombe* lifespan.

### 2.7 Maf1 phosphorylation regulates tRNA levels

Maf1 phosphorylation regulates its activity in tRNA repression in various organisms. Therefore, we investigated whether Maf1‐dependent lifespan regulation correlates with tRNA levels. Indeed, *maf1‐7E* mutant cells had defects in tRNA repression, resulting in increased level of pretRNA^Ser01‐Met01^ when compared to wild‐type cells (Figure 3c). Similar effects were also observed with pre‐tRNA^Leu06^ and pre‐tRNA^Arg05^ levels (Figure 3c). In contrast, *maf1‐7A* mutant cells showed a substantial decrease in the expression of pre‐tRNA^Ser01‐Met01^, pre‐tRNA^Leu06^, and pretRNA^Arg05^ (Figure 3c). These results suggest that Maf1 phosphorylation regulates tRNA transcription in *S. pombe*. We also measured pre‐tRNA levels in TORC1, PP2A, and PP4 mutant cells. Consistent with Maf1 phosphorylation status, *torc1* cells showed a significant decrease in pre‐tRNA levels (Figure 3d), whereas *ppa1*∆, *ppa2*∆, and *pph3*∆ cells had increased levels of pre‐tRNA (Figure 3d, e). Taken together, our results indicate that dephosphorylated Maf1 represses tRNA transcription. Our results also suggest that Maf1 phosphorylation shortens lifespan, with an exception that *pph3*∆ cells have a slightly longer lifespan compared with wild‐type cells (Figure 2d).

Our results are consistent with the idea that Maf1‐mediated tRNA transcriptional repression affects lifespan. Consistently, Pol III inhibition extends lifespan in budding yeast, worms, and fruit flies (Filer et al., 2017). To directly evaluate the role of tRNA expression in lifespan regulation in fission yeast, we used the *sfc3‐1* allele, which has a mutation in TFIIIC subunit Sfc3, leading to a reduction in Pol III activity (Iwasaki, Tanaka, Tanizawa, Grewal, & Noma, 2010). Interestingly, *sfc3‐1 maf1*∆ cells initially showed growth defects in glucose‐limited medium. However, as we culture cells for longer periods, the *sfc3‐1* mutation reversed the short life of *maf1*∆ cells (Figures 3f and S5a). These results suggest that elevated Pol III‐dependent transcription has negative impact on longevity in *maf1*∆ cells.

### 2.8 Protein synthesis elevated in *maf1*∆ cells is not a cause of lifespan shortening

**Figure 4:**
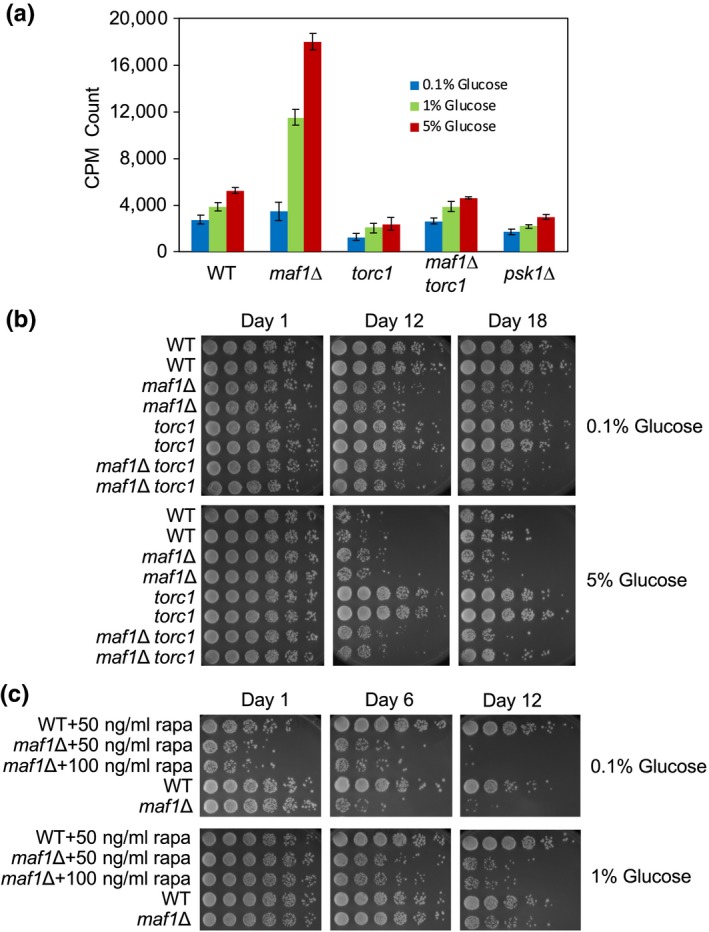
Maf1 is a major target of TORC1 in lifespan regulation. (a) Cells of the indicated genotypes were grown at 30°C in YES liquid medium supplemented with the indicated concentrations of glucose. Cells were then incubated with Trans‐^35^S for 15 min, and protein synthesis rate was expressed as the amount of radioactivity (counts per minute, CPM) incorporated in proteins. Error bars represent *SEM* obtained from three independent experiments. (b) WT, *maf1*Δ, *torc1*, and *maf1*Δ *torc1* cells were cultured in YES liquid medium with different percentages of glucose at 25°C for the indicated days, and viability was assayed on YES agar medium containing 3% glucose at 25°C. (c) WT and *maf1*Δ cells were cultured in YES medium with different percentages of glucose in the presence or absence of rapamycin. Cultures were kept at 30°C for the indicated days, and viability was assayed as described in (b) at 30°C

TORC1 activates protein translation in response to nutrient availability. Because protein synthesis is an energetically costly process, which also leads to the generation of reactive oxygen species, inhibiting protein synthesis is thought to extend lifespan (Hands, Proud, & Wyttenbach, 2009). *Drosophila* and *C. elegans maf1* mutants display increased body mass with increased protein amounts probably due to the elevated level of tRNAs (Khanna et al., 2014; Rideout et al., 2012). A recent report also suggested that the longevity increased by Pol III inhibition was likely due to the reduced levels of protein synthesis, although it was not experimentally tested (Filer et al., 2017). These results prompted us to determine the rate of protein synthesis in *S. pombe maf1*∆ cells. Protein synthesis rates were elevated in the absence of Maf1 (Figure 4a), suggesting that tRNA elevation promotes protein synthesis. In contrast, *torc1* mutant cells displayed a significant reduction in protein synthesis as expected from previous studies in other organisms (Figure 4a).

In order to address the role of increased protein synthesis in lifespan shortening in *maf1*∆ cells, we measured the rate of protein synthesis in *maf1*∆ *torc1* double‐mutant cells. As shown in Figure 4a, a *torc1* mutation abolished the increased protein synthesis in *maf1*∆ cells (*maf1*∆ *torc1*). However, TORC1 downregulation by the *torc1* mutation failed to rescue the short lifespan of *maf1*∆ cells (Figure 4b). We also treated *maf1*∆ cells with rapamycin to inhibit TORC1. However, rapamycin failed to extend the short lifespan of *maf1*∆, whereas rapamycin efficiently extended wild‐type lifespan (Figure 4c).

In *S. pombe*, Psk1, the S6K1 homolog, is controlled by TORC1 in response to nutrient availability (Nakashima et al., 2012). Because S6K1 promotes protein synthesis in various organisms (Zoncu et al., 2011), we examined the effect of *psk1* deletion on protein synthesis in *S. pombe*. As shown in Figure 4a, *psk1*∆ cells had reduced levels of protein synthesis when compared to wild‐type cells; however, *psk1*∆ cells failed to show lifespan extension (Figure S5b). Thus, our results are consistent with the notion that elevated protein synthesis is not a cause of lifespan shortening in *maf1*∆ cells. Our results also suggest that Maf1 is required for lifespan extension by TORC1 downregulation and that Maf1 functions as a major downstream regulator of TORC1 in lifespan regulation.

### 2.9 Maf1 prevents genomic instability to extend lifespan

**Figure 5:**
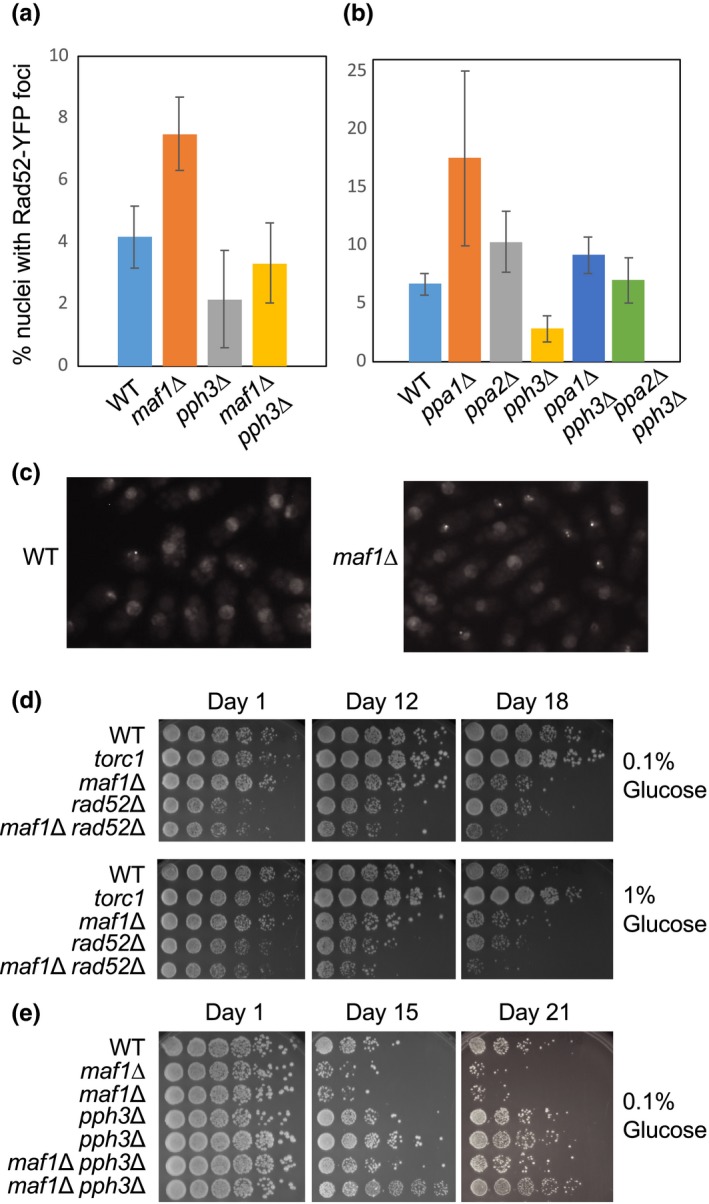
Maf1 cells accumulate DNA damage causing lifespan shortening. (a, b) The indicated cells expressing Rad52‐YFP were grown to mid‐log phase in YES liquid medium with 0.1% glucose at 25°C. The cells were then subjected to fluorescence microscopy. At least 200 cells were counted for each strain. The percentages of nuclei with at least one Rad52‐YFP focus are shown. Error bars correspond to *SEM* obtained from three independent experiments. (c) Representative microscopic images of the indicated cells are shown. (d, e) Cells of the indicated genotypes were cultured in YES liquid medium with different percentages of glucose (0.1% and 1%) at 25°C (in d) or 30°C (in e) for the indicated days. Lifespan assay was performed by plating fivefold serial dilutions of cells on YES agar medium containing 3% glucose. The agar plates were photographed after 3–5 days of incubation at 25°C (in d) or 30°C (in e)

Because the elevated protein synthesis in *maf1*∆ cells failed to affect lifespan, we sought other possible causes of lifespan shortening in these cells. High levels of transcription can cause genomic instability (Gadaleta & Noguchi, 2017). Therefore, we hypothesized that elevated tRNA transcription leads to increased levels of DNA damage in *maf1*∆ cells. Accordingly, we monitored the formation of Rad52 DNA repair foci. Rad52 binds single‐stranded DNAs at sites of DNA damage and is required for DNA repair (Lisby, Mortensen, & Rothstein, 2003). We observed significantly elevated levels of Rad52‐YFP foci formation in *maf1*∆ cells than in wild‐type cells under calorie‐restricted conditions (Figure 5a,c). There was no significant difference between wild‐type and *maf1*∆ in a high‐calorie condition although DNA damage levels were generally elevated in this condition (Figures 5a and S6). These results indicate that Maf1 alleviates DNA damage under calorie‐restricted conditions. We hypothesized that such an increase in genomic instability shortens lifespan. To test this hypothesis, we deleted *rad52* to downregulate DNA repair activity. The lifespan of *rad52* cells was shorter than wild‐type cells (Figure 5d). Intriguingly, *maf1*∆ *rad52*∆ double‐mutant cells had a much shorter lifespan than either single‐mutant cells (Figure 5d), suggesting that DNA damage accumulated in *maf1*∆ cells may undergo Rad52‐dependent DNA repair processes and that failure in these processes contributes to lifespan shortening.

### 2.10 Pph3 deletion alleviates DNA damage foci formation and extends lifespan

It is noteworthy that *pph3*∆ cells reproducibly displayed a slightly increased lifespan compared with wild‐type cells (Figure 2d). We also generated *pph3*Δ *ppa1*Δ and *pph3*Δ *ppa2*Δ double mutants and tested their lifespan. Strikingly, *pph3*∆ rescued the short lifespan of *ppa1*∆ and *ppa2*∆ cells (Figure 2d), suggesting the role of Pph3 in lifespan shortening. Pph3/PP4 is known to dephosphorylate multiple substrates including histone H2A/H2AX and Rad53/Chk2 in *S. cerevisiae* and human cells (Chowdhury et al., 2008; Keogh et al., 2006; O'Neill et al., 2007). In the absence of Pph3/PP4, cell cycle checkpoint activities persist due to elevated H2A and Rad53 phosphorylation, leading to an increased DNA repair capacity. Consistently, *pph3*∆ also suppresses hydroxyurea sensitivity and replication fork instability of *mec1‐100* cells that have a mutation in the *S. cerevisiae* cell cycle checkpoint kinase ATR (Hustedt et al., 2015). Thus, Pph3/PP4 plays an important role in completing checkpoint processes once DNA damage is repaired. Considering that Pph3 loss augments DNA repair capacity, we hypothesized that Pph3 downregulation alleviates genomic instability generated by loss of Ppa1 or Ppa2, resulting in lifespan extension. To test this hypothesis, we monitored Rad52‐YFP foci in *ppa1*∆, *ppa2*∆, and *pph3*∆ cells. As shown in Figure 5b, *ppa1*∆ and *ppa2*∆ cells had elevated levels of Rad52‐YFP focus formation (Figure 5b). In contrast, although the effect is not statistically significant, *pph3*∆ cells had a trend to display lower levels of Rad52‐YFP foci than wild‐type cells (Figure 5a,b).

These findings correlate with their lifespan; *ppa1*∆ and *ppa2*∆ cells display a shorter lifespan, whereas *pph3*∆ cells have a longer lifespan. Most significantly, we observed reduced levels of Rad52‐YFP foci formation in *ppa1*∆ *pph3*∆ and *ppa2*∆ *pph3*∆ double‐mutant cells when compared to *ppa1*∆ or *ppa2*∆ single deletion mutant cells (Figure 5b).

We then investigated whether *pph3* deletion also alleviates DNA damage accumulated in *maf1*∆ cells. Indeed, *maf1*∆ *pph3*∆ double‐mutant cells had significantly reduced levels of Rad52‐YFP foci formation than in *maf1*∆ cells (Figure 5a). Importantly, *maf1*∆ *pph3*∆ cells displayed a longer lifespan than *maf1*∆ cells (Figure 5e). These results are consistent with a notion that *pph3* deletion alleviates DNA damage, thereby promoting lifespan extension.

### 2.11 Maf1 prevents DNA damage at tRNA genes

Considering that tRNAs are highly expressed, we hypothesized that tRNA loci are prone to DNA damage, resulting in genomic instability. Studies have shown that tRNA loci are enriched with DNA damage markers such as γH2AX and Rad52 in *S. pombe* (Rozenzhak et al., 2010; Zhou et al., 2013). It is also reported that high rates of transcription cause replication fork pausing in *S. pombe* (Sabouri, McDonald, Webb, Cristea, & Zakian, 2012). Therefore, we monitored Rad52 recruitment at tRNA genes in low‐ and high‐calorie conditions, in the presence or absence of Maf1. Accordingly, we performed Chromatin immunoprecipitation analysis of Rad52 fused to 12 tandem copies of the 12Pk epitope. The Rad52‐12Pk protein is functional (Gadaleta et al., 2016) and expressed at endogenous levels from its own promoter at the *rad52* locus.

**Figure 6:**
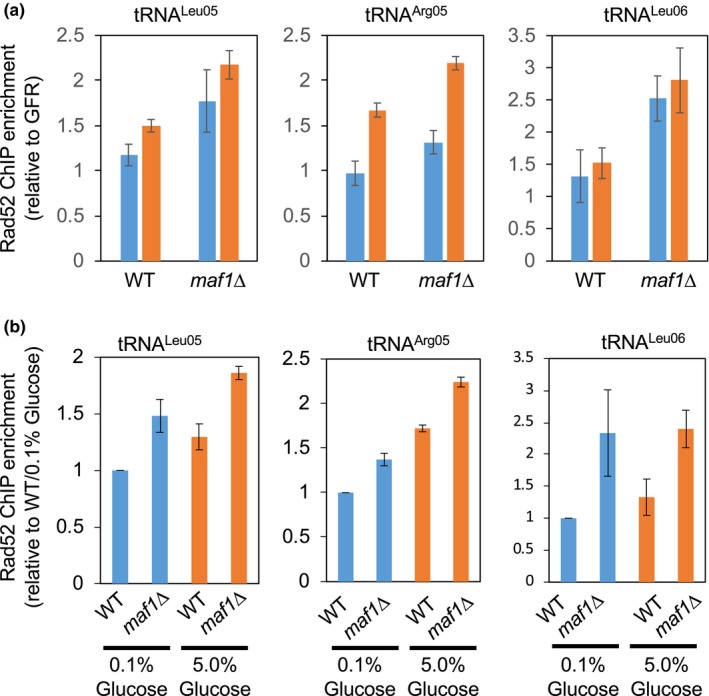
Maf1 prevents DNA damage at tRNA genes. (a, b) ChIP assays showing Rad52 enrichment at the indicated tRNA genes in wild‐type and *maf1*∆ cells. Rad52‐12Pk was chromatin‐immunoprecipitated from the indicated cells, and associated DNA was analyzed by competitive multiplex PCR to amplify DNA sequences from the indicated tRNA gene and a gene‐free region (GFR). GFR was used as an internal PCR amplification control as described in our previous study (Gadaleta et al., 2016). In (a), chromatin association of Rad52‐12Pk at the tRNA genes was presented as relative enrichment over the association at GFR. Blue bars indicate relative enrichments of Rad52 in the presence of 0.1% glucose, while orange bars show Rad52 enrichments in the presence of 5% glucose. Data are expressed as the mean of three independent experiments, and error bars correspond to *SEM*. In (b), Rad52‐12Pk association was presented as relative enrichment over the enrichment in wild‐type cells grown in the presence of 0.1% glucose

When wild‐type cells were grown under a calorie‐restricted condition, Rad52‐12Pk was not enriched at tRNA genes (tRNA^Leu05^, tRNA^Arg05^, and tRNA^Leu06^) compared with a control locus (GFR: gene‐free region; Figure 6a). In contrast, in the absence of Maf1, significant Rad52‐12Pk enrichment was observed (Figure 6a). When we monitored Rad52‐12Pk enrichment under a high‐calorie condition, two of the three tRNA genes showed enrichment compared with the control locus (Figure 6a). We then calculated relative Rad52 enrichment by normalizing to enrichment in wild‐type cells cultured under a calorie‐restricted condition (Figure 6b). Again, we observed Rad52 enrichment at the tRNA^Leu05^ and tRNA^Arg05^ tRNA genes when wild‐type cells grown in high‐calorie conditions than in calorie‐restricted medium (Figure 6b). Importantly, Rad52‐12Pk enrichment was enhanced at all the three tRNA genes in *maf1*∆ cells than in wild‐type cells. Thus, our results indicate that Maf1 prevents DNA damage at tRNA genes.

## 3 DISCUSSION

### 3.1 Maf1 is a major lifespan regulator downstream of TORC1

When nutrients are plentiful, the TORC1 pathway promotes cell growth by coordinating protein synthesis, ribosome biogenesis, transcription, and cellular metabolism. While TORC1 signaling promotes protein translation via phosphorylation of S6K and eIF4E‐BP (Zoncu et al., 2011), TORC1 may also elevate protein synthesis by increasing tRNA transcription via phosphorylation thus inactivation of Maf1, the master inhibitor of Pol III‐dependent transcription (Michels, 2011). Indeed, loss of Maf1 elevates protein synthesis likely due to increased tRNA synthesis (Figure 4a). Both protein and tRNA synthesis require considerable energy expenditure and cellular resources. Under calorie‐restricted conditions, the cell or organism must reduce its metabolism, thereby conserving its energy for survival. Accordingly, calorie restriction leads to decreased TOR signaling, which results in downregulation of protein synthesis (Zoncu et al., 2011). Therefore, TOR inhibition may conserve energy expenditure and cellular resources by decreasing protein synthesis and tRNA transcription, which may result in lifespan extension (Hands et al., 2009). However, *torc1* mutation failed to extend lifespan when Maf1 is inactivated (Figure 4b), suggesting that elevated protein synthesis observed in *maf1*∆ cells does not significantly contribute to the short‐lived phenotype of *maf1*∆ cells in *S. pombe*. Consistently, cells deleted of Psk1, the *S. pombe* S6K homolog, also failed to show lifespan extension (Figure S5b), although they displayed decreased protein synthesis levels (Figure 4a). Therefore, it is possible that, at least in *S. pombe*, decreased protein synthesis may not be a major cause of lifespan extension when TOR is downregulated in calorie‐restricted conditions.

Nevertheless, TORC1 inhibition failed to rescue the shortened lifespan of *maf1*∆ cells (Figure 4b,c). Thus, our results are consistent with the notion that Maf1 is a major lifespan regulator downstream of TORC1 in *S. pombe*.

It is important to note that Maf1’s roles in lifespan regulation vary in different organisms. Maf1^−/−^ mice are resistant to diet‐induced obesity and fatty liver diseases. These health benefits appeared to be due to decreased food intake and altered insulin signaling. In addition, Maf1^−/−^ mice have elevated autophagy, leading to lifespan extension (Bonhoure et al., 2015). Similarly, *C. elegans* deleted for *mafr‐1*, a Maf1 ortholog, showed elevated stress resistance, leading to lifespan extension under calorie‐restricted conditions (Cai & Wei, 2016). Interestingly, in the budding yeast *S. cerevisiae*, stress response mechanisms, including autophagy and mitochondrial retrograde response, were not elevated in *maf1*∆ cells (Cai & Wei, 2016). Therefore, in mice and *C. elegans*, elevated stress resistance may compensate for the lifespan‐shortening effects caused by loss of Maf1 or *mafr‐1*.

### 3.2 Role of Maf1 in lifespan regulation

How might Maf1 extend lifespan in yeast? Maf1 inhibits Pol III transcription under calorie‐restricted conditions. This results in significant decrease in tRNA and 5S rRNA synthesis, which are both energetically costly. Considering that tRNAs and 5S rRNAs account for more than 15% of total RNA (Moir & Willis, 2013), it is straightforward to suggest that Pol III inhibition saves energy expenditure and cellular resources. Consistent with this notion, limiting Pol III is shown to extend lifespan in budding yeast, worms, and fruit flies (Filer et al., 2017).

Pol III activity is influenced by the phosphorylation status of Maf1. Our mutational analyses demonstrated that a phosphomimetic form of Maf1 leads to lifespan shortening, while unphosphorylatable Maf1 extends lifespan (Figure 3b). This is also consistent with our finding that *ppa1*∆ and *ppa2*∆ cells, which have increased levels of phosphorylated Maf1 thus elevated levels of tRNA synthesis, have a shorter lifespan than wild‐type cells (Figures 2d and 3e). However, loss of Pph3 failed to shorten lifespan although tRNA synthesis is increased (Figures 2d and 3d).

Therefore, although Pol III activity may limit longevity (Filer et al., 2017), our results suggest that energy expenditure associated with tRNA synthesis is not a major cause of lifespan shortening.

Phosphatases generally have a large number of substrates; therefore, it is highly possible that loss of phosphatases can disturb multiple cellular processes. Nevertheless, our results are consistent with the notion that *pph3* deletion exerts positive effects on lifespan, which in turn compensate the possible negative effects of elevated tRNA synthesis. Indeed, Pph3 is known to dephosphorylate H2A/H2AX and Rad53/Chk2 to resume the cell cycle once DNA damage is repaired. When these substrates are phosphorylated in the absence of Pph3, cell cycle arrest is prolonged, thus potentially increasing DNA repair capacity (Chowdhury et al., 2008; Keogh et al., 2006; O'Neill et al., 2007). Consistently, *pph3* deletion reduced levels of DNA damage foci formation in wild‐type, *ppa1*∆, *ppa2*∆, and *maf1*∆ cells (Figure 5a,b). Therefore, although Maf1 phosphorylation negatively affects lifespan, the major cause of lifespan shortening appears to be elevated levels of DNA damage caused by increased tRNA synthesis due to Maf1 inactivation.

### 3.3 Role of Maf1 in preventing genomic instability

The aging process involves several physical and metabolic changes in the cell and the organism. These changes include elevated levels of ROS and DNA damage (Freitas & de Magalhaes, 2011). Rapidly growing cells may experience elevated levels of transcription as well as DNA replication in order to support cell growth. As transcription and replication use the same template DNA, collisions between the two machineries are inevitable (Gadaleta & Noguchi, 2017). Such collisions may promote genomic instability due to replication fork stalling or damage at highly transcribed genes such as tRNAs (Dutta, Shatalin, Epshtein, Gottesman, & Nudler, 2011). Indeed, elevated levels of tRNA transcription accompany the enrichment of the Rad52 DNA repair protein at tRNA genes (Figure 6a). Because Maf1 is involved in the repression of tRNA genes, loss of Maf1 would elevate the chance of collisions between replication and transcription machineries, leading to DNA damage. We consistently observed an increased level of Rad52‐YFP foci formation in *maf1*∆ cells than in wild‐type cells (Figure 5a). We also demonstrated that Rad52 is enriched at tRNA genes in *maf1*∆ cells than in wild‐type cells (Figure 6). Furthermore, *maf1*∆ *rad52*∆ double mutants showed further lifespan shortening when compared to either of the single mutants (Figure 5d). Therefore, our results are consistent with the notion that elevated genomic instability at tRNA genes is a cause of lifespan shortening in *maf1*∆ cells.

In budding yeast, *maf1*∆ cells are sensitive to replication stressing agents (Nguyen et al., 2010). In the absence of the functional DNA replication checkpoint, Maf1 is hyperphosphorylated, thus inactivated, suggesting the involvement of this checkpoint in suppressing tRNA transcription when the replication fork collides with transcription machinery at tRNA genes (Nguyen et al., 2010). This mechanism appears to be important for preventing genomic instability caused by conflicts between DNA replication and transcription, and such conflicts may also induce accelerated aging processes. Replication fork stalling is also relevant in the context of senescence. Both DNA repair and replication place strong metabolic demand on cells due to the requirement for nucleotides in these processes. Accordingly, depletion of nucleotide pools through ATM activation appears to contribute to senescence during periods of replication stress in human cells (Aird et al., 2015). Future investigation would explain the mechanism for resolving conflicts between transcription and replication in order to extend lifespan.

In summary, our investigation revealed that Maf1 is a critical mediator of lifespan regulation in *S. pombe*. Detailed investigations of how Maf1 regulates aging processes may contribute to the understanding of the therapeutic potential of Maf1 to modulate lifespan and improve late‐life function.

## 4 MATERIALS AND METHODS

### 4.1 *Schizosaccharomyces pombe* strains, plasmids, and general techniques

The *S. pombe* strains used in this study were constructed using standard techniques (Alfa, Fantes, Hyams, McLeod, & Warbrick, 1993), and their genotypes and sources are listed in Table S1. Gene deletion, gene tagging, and plasmid construction were performed as described in Supplemental Materials and Methods.

*Schizosaccharomyces pombe* cells were grown in yeast extract + supplements (YES) medium (0.5% yeast extract, 3% glucose and 2% agar supplemented with 1 × adenine, histidine‐HCL, leucine, and uracil) at 25°C or 32°C, according to their temperature sensitivity. Cells were also cultured in YES liquid medium under varying glucose concentrations (0.1%, 0.5%, 1%, 3%, and 5%) at 25°C or 30°C. The methods used for basic genetic and biochemical analysis of *S. pombe* have also been described previously (Alfa et al., 1993). Protein extract preparation, immunoblotting analysis, and fluorescence microscopy are described in Supplemental Materials and Methods.

### 4.2 Serial dilution growth assay for lifespan assessment

Cells were grown in an overnight culture until the O.D. reached approximately 1.0, where cells are in the exponential growth phase. These cells were considered to be at Day 1 of their lifespan. Cells were counted, and the cell density was adjusted to 2 × 10^7^ cells/ml with YES medium (with appropriate glucose concentration). Fivefold serial dilutions of the cell density‐adjusted cultures were prepared in a 96‐well plate and transferred to YES solid medium using a 48‐pin replicator (Sigma‐Aldrich). The YES plates were then incubated for 3–5 days at an appropriate temperature until colonies appeared. The starting *S. pombe* cell culture was returned to the incubator for continuing incubation and plated as described above at different days in order to assess the lifespan. Since auxotrophic markers, such as *leu1‐32* and *ura4‐D18*, affect *S. pombe* lifespan, isogenic auxotrophic strains were used in each lifespan assay.

### 4.3 RNA analysis

Exponentially growing *S. pombe* cells were diluted at an O.D. of 0.2 with YES liquid medium containing appropriate percentage of glucose. Cells were grown again until the O.D. reached 0.4 to 0.5. Total RNA was isolated from 1 × 10^8^ cells using Master Pure™ Yeast RNA Purification Kit (Epicentre). Total RNA sample (5 µg) was treated with 10 U of RQ1 RNase‐free DNaseI (Promega) at 37°C for 40 min in the presence of 10 U of RNase inhibitor (Epicentre), followed by further incubation at 65°C for 10 min to inactivate DNaseI in the presence of RQ1 Stop Solution. The resultant RNA sample was subjected to RT–PCR (qScript 1‐Step SYBR Green qRT‐PCR, Quanta Biosciences) using appropriate primers and following the instructions provided by the supplier. To detect pre‐tRNAs, RT–PCR samples were run on 4% polyacrylamide gel. The gel was stained by SYBR Green and analyzed with a Storm 840 phosphorimager (GE Healthcare). Relative tRNA expression levels were normalized to *act1^+^* expression level in wild‐type cells. Relative changes in gene expression were also determined by the comparative ∆∆Ct method as described previously (Gadaleta, Iwasaki, Noguchi, Noma, & Noguchi, 2015; Schmittgen & Livak, 2008). Primers used for RT–PCR are listed in Table S2.

### 4.4 Protein synthesis assay

Protein synthesis assay was performed as described previously (Wang & Chen, 2015) with modifications. Briefly, exponentially growing *S. pombe* cells (5 × 10^6^ cells) were incubated with 5.5 µCi of Trans‐^35^S‐Label (MP Biomedicals) for 15 min at 30°C. Protein labeling was terminated by adding cycloheximide (100 µg/ml) and incubating on ice for 5 min. Cells were collected by centrifugation, washed by 20% TCA, and resuspended in 250 µl of 20% TCA. Cells were lysed by glass beads using a FastPrep cell disruptor for 20 s twice at speed 6 at 4°C. 100 µl of cell lysate was recovered, and 1 ml of 5% TCA was added to the sample. Protein precipitates were collected on GF/C filters (Whatman 1822‐025) using a Millipore Model 1225 sampling manifold. Filters were washed with 10 ml of 5% TCA and then with 10 ml of 95% ethanol. Radioactivity levels incorporated in proteins were determined in a liquid scintillation counter.

### 4.5 Chromatin immunoprecipitation assay

Chromatin immunoprecipitation assay and its quantification were performed as previously described (Gadaleta et al., 2016, 2015). Briefly, cells in mid‐log phase were fixed with 3% formaldehyde, and chromatin was shared into 500‐ to 700‐bp fragments. Rad52‐12Pk was immunoprecipitated using the mouse monoclonal anti‐V5/Pk1 antibody (AbD Serotec) in combination with Protein G‐conjugated Dynabeads (Life Technologies). DNA was extracted from the immunoprecipitates and analyzed by PCR using primers designed to amplify the indicated loci. The PCR products were then run on polyacrylamide gels, stained with SYBR Green I (Life Technologies), and analyzed with Typhoon FLA 7000 Phosphorimager (GE Healthcare). Relative enrichment of the Rad52‐associated DNA sequences was calculated by multiplex PCR including primers that amplify a control locus (GFR) as an internal control as previously described (Gadaleta et al., 2016, 2015).

## Conflict of interest

None declared.
