# Supplementary material for: Maf1‐dependent transcriptional regulation of tRNAs prevents genomic instability and is associated with extended lifespan
Source: Aging Cell. 2019 Dec 12;19(2):e13068. doi: 10.1111/acel.13068 (PMC6996946; doi:10.1111/acel.13068)
Supplement: Supplementary file 1 [file ACEL-19-e13068-s001.pdf]

Supplemental Materials and Methods

S. pombe strains and plasmids

*maf1*Δ (*maf1*::*hphMX6*, *maf1*::*kanMX6*), *pph3*Δ (*pph3*::*kanMX6*), and *psk1*Δ (*psk1*::*kanMX6*) were generated by PCR-based integration methods to replace each open reading frame with the *kanMX6* or *hphMX6* gene. *maf1-3FLAG* (*maf1-3FLAG*:*kanMX6*), and *maf1-GFP* (*maf1-GFP*:*hphMX6*) were also generated by PCR-based integration methods (Bahler *et al.* 1998; Krawchuk & Wahls 1999; Noguchi *et al.* 2008), to construct a FLAG or GFP tag at the C-terminus of each gene. Mutations and epitope-tagged genes have been previously described for *rad52*Δ (*rad52*::*hphMX6*), *swi1*Δ (*swi1*::*kanMX6*), *rad52-12Pk* (*rad52-12Pk*::*kanMX6*) (Noguchi *et al.* 2003; Gadaleta *et al.* 2016) and *sfc3-1* (Iwasaki *et al.* 2010). *ppa1*Δ (*ppa1*::*ura4*$^{+}$, FY9908), *ppa2*Δ (*ppa2*::*ura4*$^{+}$, FY9910), and *torc1* (*tor2*^{ts}[L2048S]:*kan*^{r}, FY21019) were obtained from National BioResource Project Japan.

To detect Rad52-YFP DNA repair foci, pJK210-rad52CT-YFP (Rapp *et al.* 2010) was digested by *Afl*II and inserted at the *rad52* locus of various *S. pombe* strains. pJK148-rad52CT-YFP, which also has the *rad52CT-YFP* gene (1.5-kb 3′-coding region of the *rad52* gene fused to YFP cDNA) in pJK148 (Keeney & Boeke 1994), was also constructed and used to detect Rad52-YFP foci. To construct pJK148-maf1-3FLAG, the 1.7 kb genomic fragment containing the *maf1-3FLAG* gene was amplified from genomic DNA preparation from *S. pombe maf1-3FLAG* cells and inserted into *Eco*R1/*Bam*HI site of pJK148. *maf1* mutants were generated by Kunkel site-directed mutagenesis in pJK148-maf1-3FLAG and integrated at the *leu1* locus of a *maf1*::*kanMX6* strain.

Protein extract preparation and antibodies for immunoblotting

Exponentially growing *S. pombe* cells were washed in Stop Buffer (150 mM NaCl, 50 mM NaF, 10 mM EDTA, 1 mM NaN_3, 1 mM Na_3VO_4, 5 mM N-ethylmaleimide), resuspended in 100 µL Stop Buffer, boiled for 5 minutes, and chilled on ice. To lyse cells, 120 µL 2 × SDS-PAGE-8 M

Urea sample buffer (150 mM Tris-HCL, pH 6.8, 6% SDS, 6 mM EDTA, 30% glycerol, 8 M urea, 1 mM Na_3VO_4, 5 mM N-ethylmaleimide, 1 mM microsistin, 0.1 mM ocadaic acid) and 0.5-mm glass beads (Biospec Product) were added to the sample and processed by a FastPrep cell disruptor (Qbiogene, Irvine, CA) for 20 seconds twice at speed 6 at 4°C. Cell lysates were recovered in a microfuge tube and clarified by centrifugation at 13,000 rpm for 10 min at 4°C. Protein concentration was determined using BCA protein Assay Reagent (Thermo Fisher Scientific, Waltham, MA). Protein extracts were boiled for 5 min in the presence of 5% beta-mercaptoethanol, and the protein concentrations were equalized.

For immunoblotting, FLAG fusion proteins were probed with the anti-FLAG M2 monoclonal antibody (Sigma-Aldrich); and TAT1 [gift from Dr. Keith Gull (Woods *et al.* 1989)] to detect tubulin.

Immunoprecipitation and phosphatase treatment of the MAF1-5FLAG protein

Cells expressing Maf1-5FLAG were cultured in YES medium supplemented with 3% glucose, collected, and suspended in lysis buffer A (50 mM Tris-HCl pH8.0, 150 mM NaCl, 0.1% NP-40, 10% glycerol, 5 mM EDTA, and 5 mM *N*-methylmaleimide) supplemented with phosphatase inhibitors (50 mM NaF, 1 mM Na_3VO_4, 1 μM microcystin, 0.1 μM okadaic acid), 0.2 mM *p*-4-amidoinophenyl-methane sulfonyl fluoride hydrochloride monohydrate, and Halt protease inhibitor cocktail (Thermo Fisher Scientific). Cells were they lysed with glass beads using a FastPrep cell disruptor as described above. Protein extracts were then clarified by centrifugation at 13,000 rpm for 10 min at 4°C, mixed with anti-FLAG MS agarose (Sigma-Aldrich), incubated for 2 hours at 4˚C. The agarose beads were collected, washed three times in lysis buffer A without phosphatase inhibitors. Maf1-5FLAG on agarose beads was treated with 200 units of λ phosphatase (New England Biolabs) with or without phosphatase inhibitors (50 mM NaF, 10 mM Na_3VO_4, 1 μM okadaic acid, 4 μM microcystin) and processed for Western blotting using anti-FLAG polyclonal antibody (F7425, Sigma-Aldrich).

Fluorescence microscopy

Detection of Rad52-YFP has been described previously (Noguchi *et al.* 2009; Gadaleta *et al.* 2016). Cells are grown to mid-log phase at 25˚C. The quantification of Rad52-YFP protein was performed at least three times, and at least 200 cells were counted for each experiment.

SUPPLEMENTARY FIGURE LEGENDS

Supplementary Figure S1:

(A) WT cells were cultured overnight at 30°C in YES liquid medium containing different percentages of glucose (0.1, 0.5, 1, 3, and 5%). Exponentially growing cells were diluted to OD_{600} of 0.5 at the starting point (0 hr) and incubated at 30˚C. OD_{600} was determined at the indicated times.

(B) WT and *maf1*Δ cells were grown at 30°C in YES liquid medium containing different percentages of glucose, OD_{600} and was monitored at the indicated times.

(C) *maf1*Δ cells transformed with the control vector or Maf1-expressing vector were evaluated in the lifespan assay described in Figure 1C. Representative images of repeat experiments are shown.

Supplementary Figure S2:

The cells of the indicated genotypes were first cultured at 30°C in YES liquid medium with different percentages of glucose (0.1, 1 and 5%) for the indicated days. Five-fold serial dilutions of the cells were then plated on YES agar medium containing 3% glucose, incubated for 3 days at 30˚C, and photographed.

Supplementary Figure S3:

(A) Immunoprecipitated Maf1–FLAG was incubated with or without λ phosphatase and detected by Western blotting. The panel also shows Maf1–FLAG treated with λ phosphatase in the presence of phosphatase inhibitors.

(B, C, D) Cells of the indicated genotypes were engineered to express Maf1–5FLAG and grown at 30˚C in YES liquid medium supplemented with indicated percentages glucose. Protein extracts were prepared, and Western blotting of Maf1-5FLAG was performed. Tubulin was used as a loading control. Representative images of repeat experiments are shown.

Supplementary Figure S4:

(A) Schematic drawing of Maf1 homologs from *S. cerevisiae*, humans and *S. pombe*. Yellow boxes indicate the regions of amino acid sequences that are highly conserved among species. Potential phosphorylation sites are also indicated.

(B) ClustalW multiple sequence alignment of Maf1 proteins from humans, *S. cerevisiae*, and *S. pombe*.

Supplementary Figure S5:

(A) The *sfc3-1* mutation rescues the short lifespan of *maf1*Δ cells.

(B) *psk1*Δ cells failed to show a lifespan shortening phenotype.

In *A* and *B*, cells of the indicated genotypes were first grown at 30˚C for the indicated days in YES liquid medium supplemented with 0.1%, 1% or 5% glucose. To evaluate cell viability, five-fold serial dilutions of the cells were prepared and plated on YES agar medium supplemented with 3% glucose. The agar plates were photographed after 3 days of incubation at 30˚C.

Supplementary Figure S6:

Rad52-YFP foci formation in normal glucose medium. WT and *maf1*Δ cells expressing Rad52-YFP were grown to mid-log phase in YES liquid medium with 3 % glucose at 25˚C for the indicated days. The cells were then subjected to fluorescence microscopy. Quantification of Rad52-YFP was performed as described in Figure 7A legend. There was no significant difference between WT and *maf1*Δ cells in Rad52-YFP formation.

Supplementary Table S1: *S. pombe* strains used in this study.

Supplementary Table S2: Oligonucleotide primers used in this study.

Supplementary References

Bahler J, Wu JQ, Longtine MS, Shah NG, McKenzie A, 3rd, Steever AB, Wach A, Philippsen P, Pringle JR (1998). Heterologous modules for efficient and versatile PCR-based gene targeting in Schizosaccharomyces pombe. *Yeast*. **14**, 943-951.

Gadaleta MC, Das MM, Tanizawa H, Chang YT, Noma K, Nakamura TM, Noguchi E (2016). Swi1Timeless Prevents Repeat Instability at Fission Yeast Telomeres. *PLoS Genet*. **12**, e1005943.

Iwasaki O, Tanaka A, Tanizawa H, Grewal SI, Noma K (2010). Centromeric localization of dispersed Pol III genes in fission yeast. *Molecular biology of the cell*. **21**, 254-265.

Keeney JB, Boeke JD (1994). Efficient targeted integration at leu1-32 and ura4-294 in Schizosaccharomyces pombe. *Genetics*. **136**, 849-856.

Krawchuk MD, Wahls WP (1999). High-efficiency gene targeting in Schizosaccharomyces pombe using a modular, PCR-based approach with long tracts of flanking homology. *Yeast*. **15**, 1419-1427.

Noguchi C, Garabedian MV, Malik M, Noguchi E (2008). A vector system for genomic FLAG epitope-tagging in Schizosaccharomyces pombe. *Biotechnol J*. **3**, 1280-1285.

Noguchi E, Ansbach AB, Noguchi C, Russell P (2009). Assays used to study the DNA replication checkpoint in fission yeast. *Methods Mol Biol*. **521**, 493-507.

Noguchi E, Noguchi C, Du LL, Russell P (2003). Swi1 prevents replication fork collapse and controls checkpoint kinase Cds1. *Mol Cell Biol*. **23**, 7861-7874.

Rapp JB, Noguchi C, Das MM, Wong LK, Ansbach AB, Holmes AM, Arcangioli B, Noguchi E (2010). Checkpoint-dependent and -independent roles of Swi3 in replication fork recovery and sister chromatid cohesion in fission yeast. *PLoS One*. **5**, e13379.

Woods A, Sherwin T, Sasse R, MacRae TH, Baines AJ, Gull K (1989). Definition of individual components within the cytoskeleton of Trypanosoma brucei by a library of monoclonal antibodies. *J Cell Sci*. **93 (Pt 3)**, 491-500.

Supplementary Figure S1

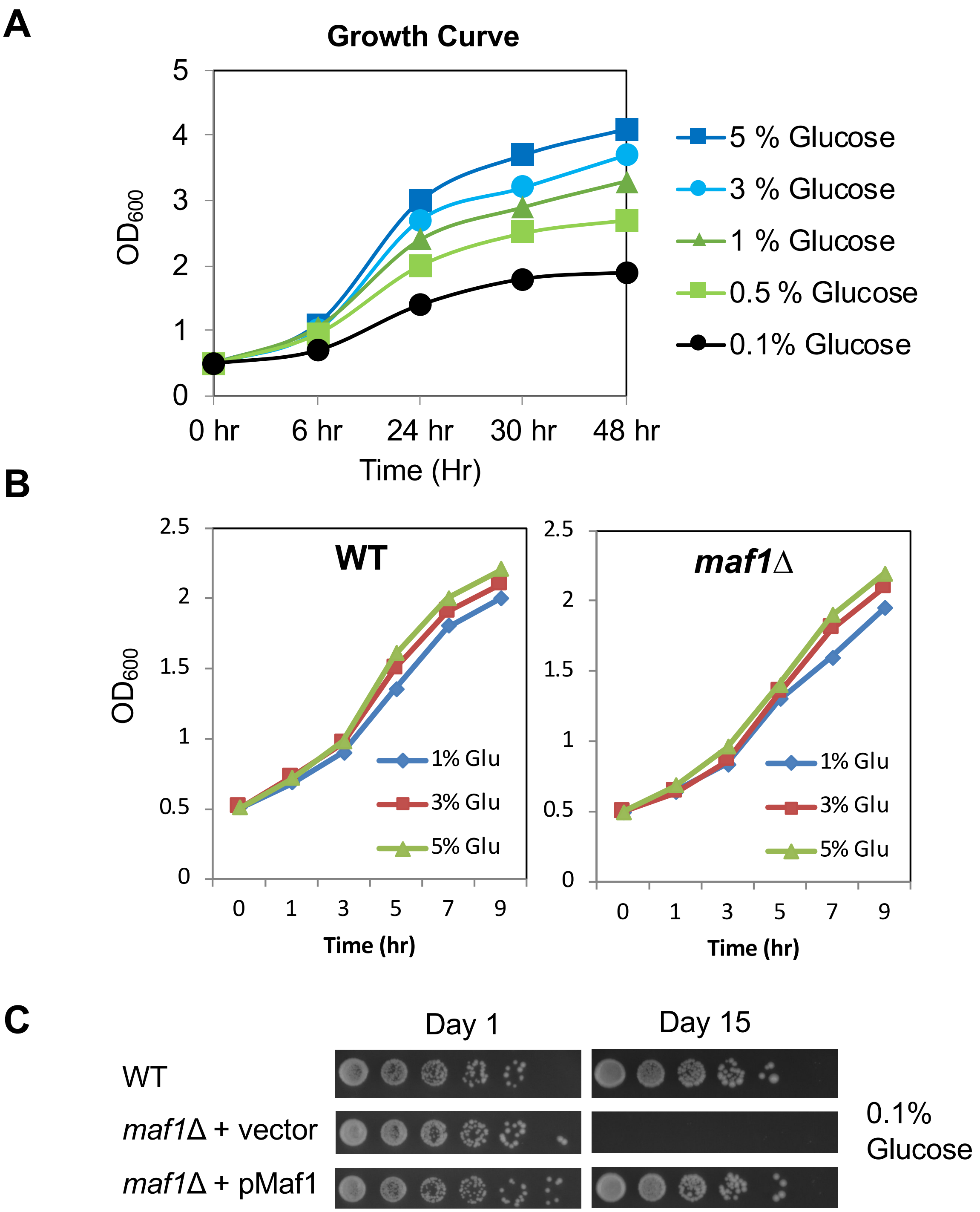

Supplementary Figure S2

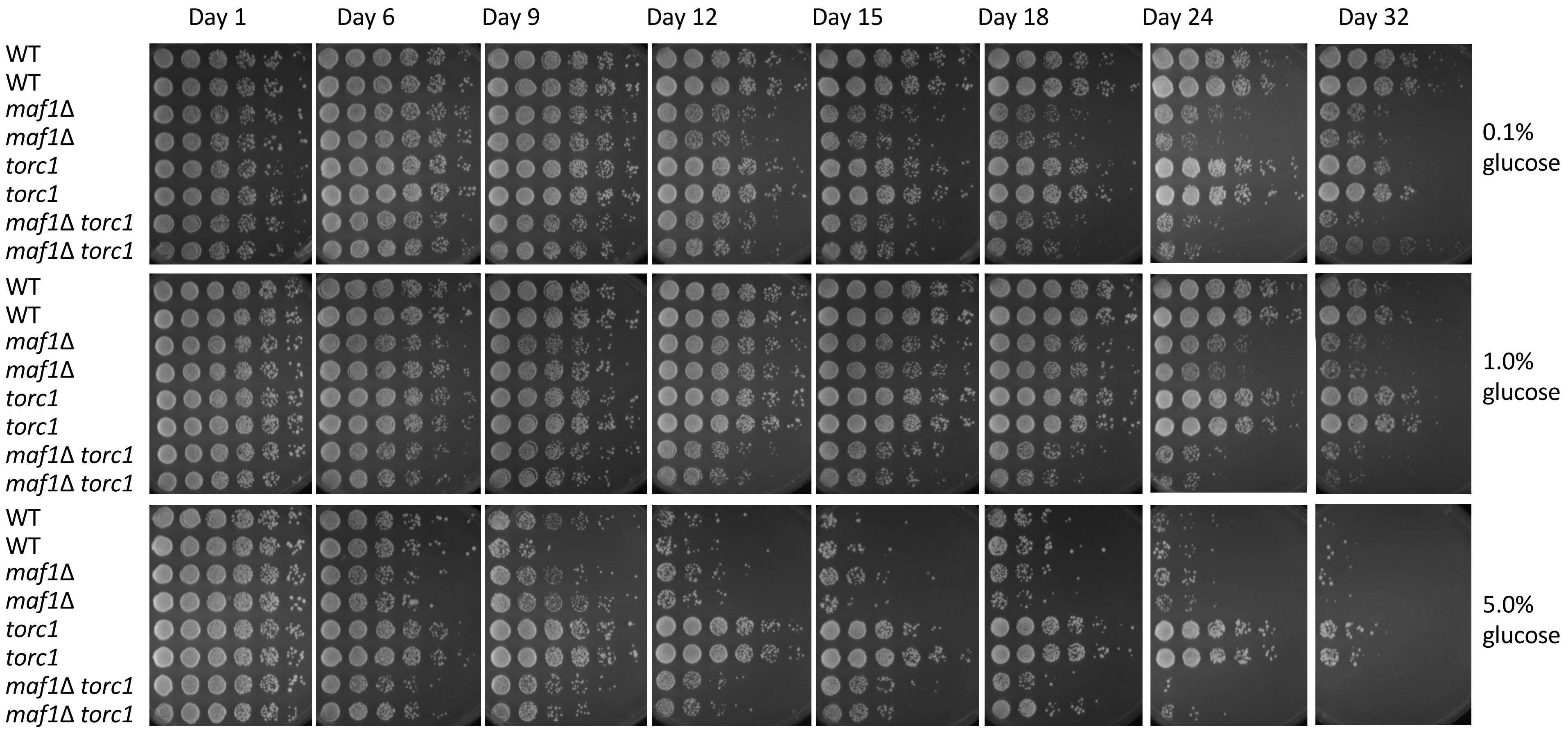

Supplementary Figure S3

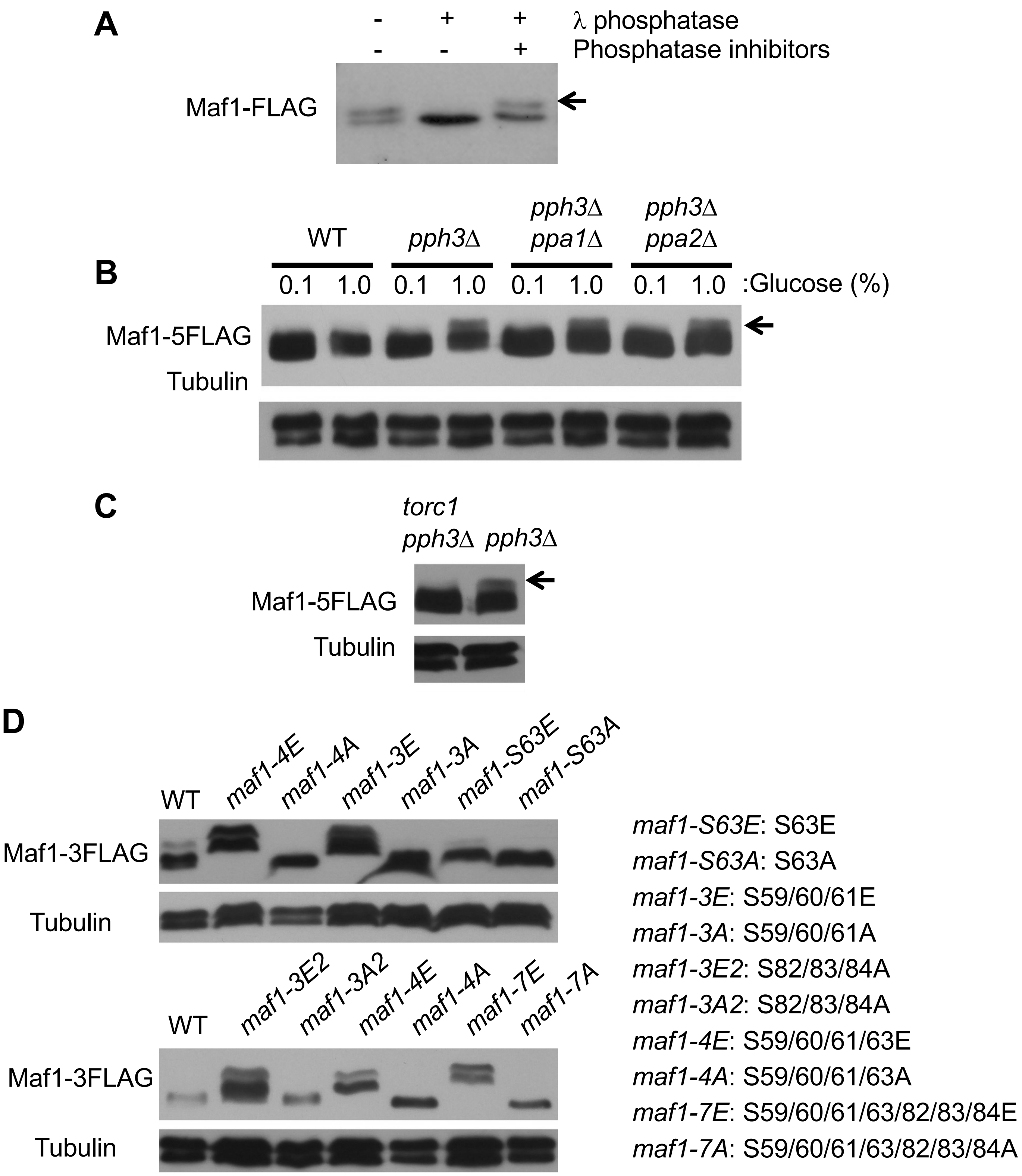

Supplementary Figure S4

A

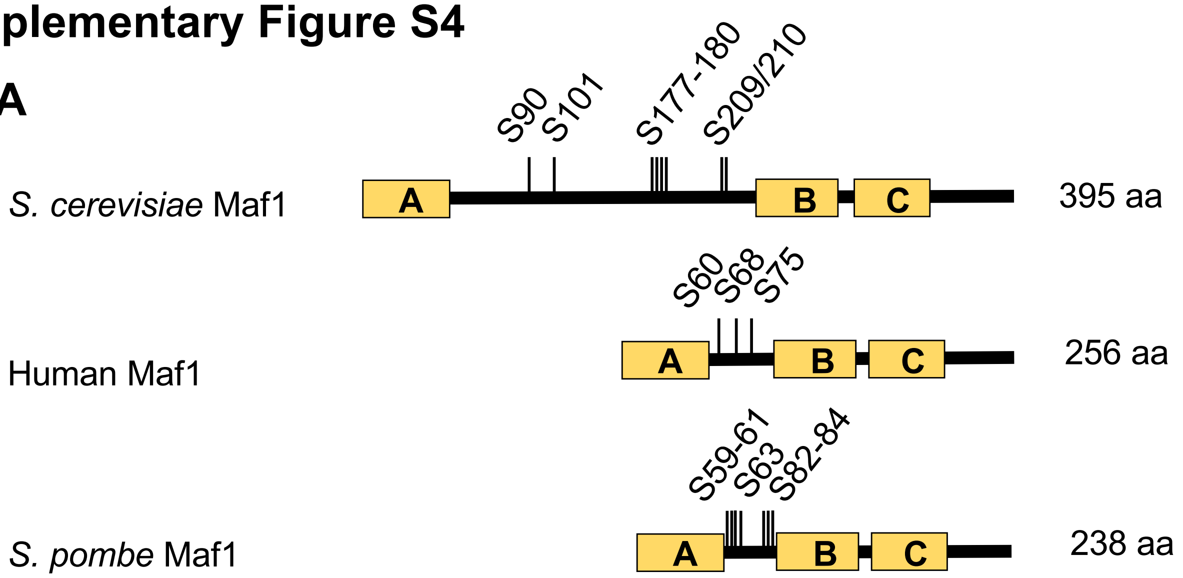

B

```
Cerevisiae    MKFIDELDIERVNQTLNFETNDCKIVGSCDIFTTKAVASDRKLYKTIDQHLDTILQENEN 60
Pombe         MKFLELADLDTVNNALSFDADDCRIRGKCELYTTKSTNSDKKLFKAIEN----------- 49
Human         MKLLENSSFEAINSQLTVETGDAHIIGRIESYSCKMAGDDKHMFKQFCQ----------E 50
              **:::  .::  :*.  *..:::.*.:* *   :  ::  *  . .*::::*  :  :

Cerevisiae    YNATLQQQLAAPETNQSPCSSPFYSNRRDSNSFWEQKRRISFSEYNSNNNTNNSNGNSSN 120
Pombe         ---RCQEDLFALSSSKSP-----------------------EY-------------- 66
Human         GQPHVLEALSPPQTSGLS---------------------------------------- 68
                    :  *  .  .:.   .

Cerevisiae    NNNYSGPNGSSPATFPKSAKLNDQNLKELVSNYDSGSMSSSSLDSSSKNDERIRRRSSSS 180
Pombe         ---------------------------------------AFSLTQQS------------- 74
Human         ---------------------------------------PSRLSKSQGGE---------- 79
                                                     .   *  ...

Cerevisiae    ISSFKSGKSSNNNYSSGTATNNVNKRRKSSINERPSNLSLGPFGPINEPSSRKIFAYLIA 240
Pombe         -----------------------------------------PFGPLDQSSSRRTFMYIVA 93
Human         -----------------------------------------EEGPLSDKCSRKTLFYLIA 98
                                                        **:.:  .**:  :  *::*

Cerevisiae    ILNASY-PDHDFSSVEPTDFVKT-SLKTFISKFENTLYSLGR----QPEEWVWEVINSHM 294
Pombe         TLNASY-PDHDFSSLQPTDFYKEPSLSRVVDSVNSTLNNIGRG--RLSVNGIWEIIDRHI 150
Human         TLNESFRPDYDFSTARSHEFSREPSLSWVVNAVNCSLFSAVREDFKDLKPQLWNAVDEEI 158
               ** *:  **:***:  ..  :*  :   **.  .:.  .:  :*  .   *          :*:  ::  .:

Cerevisiae    TLSDCVLFQYSP-SNSFLEDEPGYLWNLIGFLYNRKRKRVAYLYLICSRLNSSTGEVEDA 353
Pombe         NLSDCSVYSYTPDSDSDPYGDDALIWGMSYFFFNKNMKRMLYLS-----LHGLGKEVSGR 205
Human         CLAECDIYSYNPDLDSDPFGEDGSLWSFNYFFYNKRLKRIVFFS-----CRSISGSTYTP 213
               *::* ::.*.*   :*    .:  .  :*.:    *::*:.  **:  ::          ..    ..

Cerevisiae    LAKKPQGKLIIDDGSNEYEGEYDFTYDENVIDDKSDQEESLQ- 395
Pombe         NRYGNDDDSVFTPLADDAE---PSDFDDDWVANMDD------- 238
Human         SEAGNELDMELGEEEVEEESRSRGSGAEETSTMEEDRVPVICI 256
                  :  .   :      :  *        ::      .*
```

Supplementary Figure S5

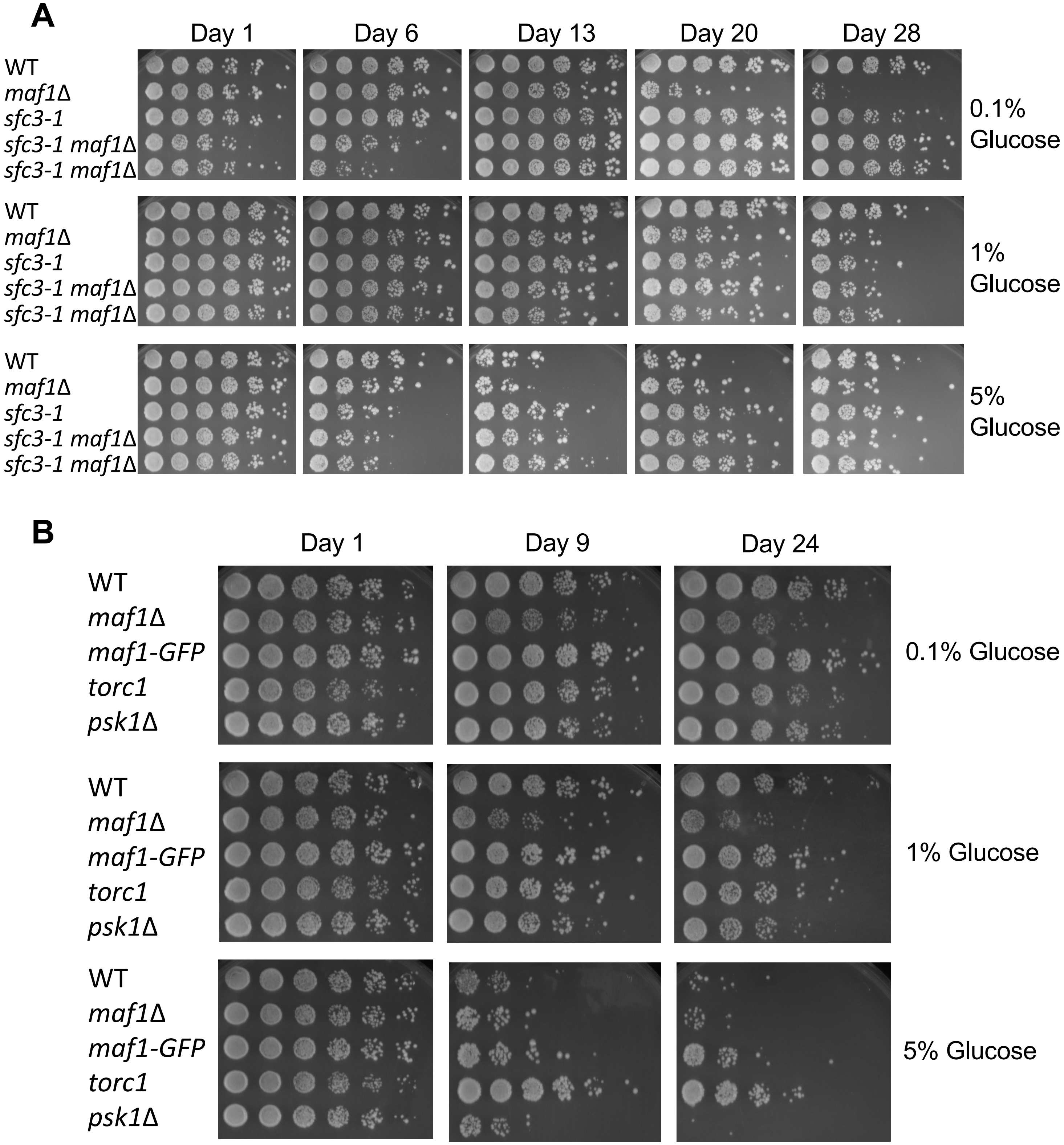

Supplementary Figure S6

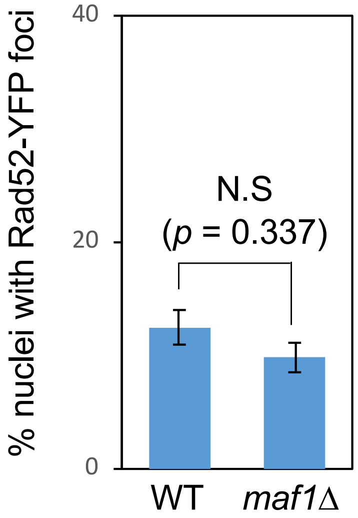

Supplemental Table S1: *S. pombe* strains used in this study

Figure	Strain	genotype
1A	Y1	h- leu1-32 ura4-D18
1A	Y3979	h- maf1::hphMX6 leu1-32 ura4-D18
1B	Y4250	h+ rad52-12PK:kanrMX6 leu1-32 ura4-D18
1B	Y5159	h+ maf1::hphMX6 rad52-12PK:kanMX6 leu1-32 ura4-D18
1B	Y5186	h+ tor2ts[L2048S]KanR rad52-12PK:KanrMX6 leu1-32 ura4-D18
1C, D	Y1	h- leu1-32 ura4-D18
1C, D	Y2	h+ leu1-32 ura4-D18
1C, D	Y3979	h- maf1::hphMX6 leu1-32 ura4-D18
1C, D	Y3980	h+ maf1::hphMX6 leu1-32 ura4-D18
2A	Y4368	h- maf1-FLAG:hphMX6 leu1-32 ura4-D18
2A	Y4380	h- maf1-FLAG:hphMX6 pph3::kanMX6 leu1-32 ura4-D18 his3-D1
2B	Y4871	h- maf1-FLAG:hphMX6 leu1-32
2B	Y4949	h- ppa1::ura4+ maf1-FLAG:hphMX6 leu1-32 ura4-D18
2B	Y4955	h- ppa2::ura4+ maf1-FLAG:hphMX6 leu1-32 ura4-D18
2C	Y2	h+ leu1-32 ura4-D18
2C	Y4782	h+ tor2ts[L2048S]KanR leu1-32 ura4-D18
2C	Y3980	h+ maf1::hphMX6 leu1-32 ura4-D18
2D	Y2312	h+ leu1-32
2D	Y4870	h+ pph3::kanMX6 leu1-32
2D	Y4905	h- ppa1::ura4+ leu1 ura4
2D	Y4906	h- ppa2::ura4+ leu1 ura4
2D	Y4951	h+ ppa1::ura4+ pph3::hphMX6 leu1-32 ura4-D18
2D	Y4952	h- ppa1::ura4+ pph3::hphMX6 leu1-32 ura4-D18
2D	Y4956	h- ppa2::ura4+ pph3::hphMX6 leu1-32 ura4-D18
2D	Y4957	h- ppa2::ura4+ pph3::hphMX6 leu1-32 ura4-D18
3A	Y4983	h- maf1::hphMX6 leu1-32:leu1+ maf1-FLAG ura4-D18
3A	Y5038	h- maf1::hphMX6 leu1-32:leu1+ maf1-S59/60/61A-FLAG (maf1-3A) ura4-D18
3A	Y5040	h- maf1::hphMX6 leu1-32:leu1+ maf1-S63A-FLAG ura4-D18
3A	Y4987	h- maf1::hphMX6 leu1-32:leu1+ maf1-S82/83/84A-FLAG (maf1-3A2) ura4-D18
3A	Y4967	h+ maf1::hphMX6 leu1-32:leu1+ maf1-S59/60/61/63A-FLAG (maf1-4A) ura4-D18
3A	Y4965	h- maf1::hphMX6 leu1-32:leu1+ maf1-S59/60/61/63/82/83/84A-FLAG (maf1-7A) ura4-D18
3B	Y4983	h- maf1::hphMX6 leu1-32:leu1+ maf1-FLAG ura4-D18
3B	Y5042	h- maf1::hphMX6 leu1-32:leu1+ maf1-S82/83/84E-FLAG (maf1-3E2) ura4-D18
3B	Y4987	h- maf1::hphMX6 leu1-32:leu1+ maf1-S82/83/84A-FLAG (maf1-3A2) ura4-D18
3B	Y5044	h- maf1::hphMX6 leu1-32:leu1+ maf1-S59/60/61/63E-FLAG (maf1-4E) ura4-D18
3B	Y4967	h+ maf1::hphMX6 leu1-32:leu1+ maf1-S59/60/61/63A-FLAG (maf1-4A) ura4-D18
3B	Y5046	h- maf1::hphMX6 leu1-32:leu1+ maf1-S59/60/61/63/82/83/84E-FLAG (maf1-7E) ura4-D18
3B	Y4965	h- maf1::hphMX6 leu1-32:leu1+ maf1-S59/60/61/63/82/83/84A-FLAG (maf1-7A) ura4-D18
3B	Y5048	h- maf1::hphMX6 leu1-32:leu1+ maf1-S59/60/61E-FLAG (maf1-3E) ura4-D18
3B	Y5038	h- maf1::hphMX6 leu1-32:leu1+ maf1-S59/60/61A-FLAG (maf1-3A) ura4-D18
3B	Y5050	h- maf1::hphMX6 leu1-32:leu1+ maf1-S63E-FLAG ura4-D18
3B	Y5040	h- maf1::hphMX6 leu1-32:leu1+ maf1-S63A-FLAG ura4-D18
3C	Y4983	h- maf1::hphMX6 leu1-32:leu1+ maf1-FLAG ura4-D18
3C	Y4965	h- maf1::hphMX6 leu1-32:leu1+ maf1-S59/60/61/63/82/83/84A-FLAG (maf1-7A) ura4-D18
3C	Y5046	h- maf1::hphMX6 leu1-32:leu1+ maf1-S59/60/61/63/82/83/84E-FLAG (maf1-7E) ura4-D18
3D	Y1	h- leu1-32 ura4-D18
3D	Y3979	h- maf1::hphMX6 leu1-32 ura4-D18
3D	Y4570	h- pph3::hphMX6 leu1-32 ura4-D18
3D	Y4783	h- tor2ts[L2048S]KanR leu1-32 ura4-D18
3E	Y2311	h- leu1-32
3E	Y4777	h- maf1::hphMX6 leu1-32
3E	Y4905	h- ppa1::ura4+ leu1 ura4
3E	Y4906	h- ppa2::ura4+ leu1 ura4
4A	Y2	h+ leu1-32 ura4-D18
4A	Y3980	h+ maf1::hphMX6 leu1-32 ura4-D18
4A	Y4782	h+ tor2ts[L2048S]KanR leu1-32 ura4-D18
4A	Y5052	h+ tor2ts[L2048S]KanR maf1::hphMX6 leu1-32 ura4-D18
4A	Y5062	h+ psk1::kanMX6 leu1-32 ura4-D18

4B	Y1	h- leu1-32 ura4-D18
4B	Y2	h+ leu1-32 ura4-D18
4B	Y3979	h- maf1::hphMX6 leu1-32 ura4-D18
4B	Y3980	h+ maf1::hphMX6 leu1-32 ura4-D18
4B	Y4782	h+ tor2ts[L2048S]KanR leu1-32 ura4-D18
4B	Y4783	h- tor2ts[L2048S]KanR leu1-32 ura4-D18
4B	Y5051	h- tor2ts[L2048S]KanR maf1::hphMX6 leu1-32 ura4-D18
4B	Y5052	h+ tor2ts[L2048S]KanR maf1::hphMX6 leu1-32 ura4-D18
4C	Y1	h- leu1-32 ura4-D18
4C	Y3979	h- maf1::hphMX6 leu1-32 ura4-D18
5A	Y5126	h+ leu1-32 rad52-YFP:leu+
5A	Y5188	h+ maf1::hphMX6 rad52-YFP:leu+ leu1-32 ura4-D18
5A	Y5115	h+ pph3::kanMX6 leu1-32 rad52-YFP:leu+
5A	Y5190	h+ pph3::kanMX6 maf1::hphMX6 rad52-YFP:leu+ leu1-32 ura4-D18
5B	Y5126	h+ leu1-32 rad52-YFP:leu+
5B	Y5124	h- ppa1::ura4+ rad52-YFP:leu+ leu1-32 ura4-D18
5B	Y5123	h- ppa2::ura4+ rad52-YFP:leu+ leu1-32 ura4-D18
5B	Y5115	h+ pph3::kanMX6 rad52-YFP:leu+ leu1-32
5B	Y5117	h+ ppa1::ura4+ pph3::hphMX6 rad52-YFP:leu+ leu1-32 ura4-D18
5B	Y5119	h- ppa2::ura4+ pph3::hphMX6 rad52-YFP:leu+ leu1-32 ura4-D18
5C	Y4627	h+ rad52-YFP:ura4+ leu1-32 ura4-D18
5C	Y4006	h+ maf1::hphMX6 rad52-YFP:ura4+ leu1-32 ura4-D18
5D	Y2	h+ leu1-32 ura4-D18
5D	Y4782	h+ tor2ts[L2048S]KanR leu1-32 ura4-D18
5D	Y3980	h+ maf1::hphMX6 leu1-32 ura4-D18
5D	Y4708	h+ rad52::hphMX6 leu1-32 ura4-D18
5D	Y4862	h+ maf1::kanMX6 rad52::hphMX6 leu1-32 ura4-D18
5E	Y2	h+ leu1-32 ura4-D18
5E	Y3979	h- maf1::hphMX6 leu1-32 ura4-D18
5E	Y3980	h+ maf1::hphMX6 leu1-32 ura4-D18
5E	Y5470	h- pph3::hphMX6 leu1-32 ura4-D18
5E	Y4571	h+ pph3::hphMX6 leu1-32 ura4-D18
5E	Y4435	h+ maf1::KanMX6 pph3::hphMX6 leu1-32 ura4-D18
5E	Y4436	h- maf1::KanMX6 pph3::hphMX6 leu1-32 ura4-D18
6A, B	Y4250	h+ rad52-12PK:kanrMX6 leu1-32 ura4-D18
6A, B	Y5159	h+ maf1::hphMX6 rad52-12PK:kanMX6 leu1-32 ura4-D18
S1A	Y1	h- leu1-32 ura4-D18
S1B, C	Y1	h- leu1-32 ura4-D18
S1B, C	Y3979	h- maf1::hphMX6 leu1-32 ura4-D18
S2	Y1	h- leu1-32 ura4-D18
S2	Y2	h+ leu1-32 ura4-D18
S2	Y3979	h- maf1::hphMX6 leu1-32 ura4-D18
S2	Y3980	h+ maf1::hphMX6 leu1-32 ura4-D18
S2	Y4782	h+ tor2ts[L2048S]KanR leu1-32 ura4-D18
S2	Y4783	h- tor2ts[L2048S]KanR leu1-32 ura4-D18
S2	Y5051	h- tor2ts[L2048S]KanR maf1::hphMX6 leu1-32 ura4-D18
S2	Y5052	h+ tor2ts[L2048S]KanR maf1::hphMX6 leu1-32 ura4-D18
S3A	Y4368	h- maf1-FLAG:hphMX6 leu1-32 ura4-D18
S3B	Y4871	h- maf1-FLAG:hphMX6 leu1-32
S3B	Y4380	h- maf1-FLAG:hphMX6 pph3::kanMX6 leu1-32 ura4-D18 his3-D1
S3B	Y4947	h- ppa1::ura4+ maf1-FLAG:hphMX6 pph3::hphMX6 leu1-32 ura4-D18
S3B	Y4953	h- ppa2::ura4+ maf1-FLAG:hphMX6 pph3::hphMX6 leu1-32 ura4-D18
S3C	Y4944	h+ maf1-FLAG:hphMX6 tor2ts[L2048S]KanR pph3::hphMX6 leu1-32 ura4-D18
S3C	Y4380	h- maf1-FLAG:hphMX6 pph3::kanMX6 leu1-32 ura4-D18 his3-D1
S3D	Y4983	h- maf1::hphMX6 leu1-32:leu1+ maf1-FLAG ura4-D18
S3D	Y5044	h- maf1::hphMX6 leu1-32:leu1+ maf1-S59/60/61/63E-FLAG (maf1-4E) ura4-D18
S3D	Y4967	h+ maf1::hphMX6 leu1-32:leu1+ maf1-S59/60/61/63A-FLAG (maf1-4A) ura4-D18
S3D	Y5048	h- maf1::hphMX6 leu1-32:leu1+ maf1-S59/60/61E-FLAG (maf1-3E) ura4-D18
S3D	Y5038	h- maf1::hphMX6 leu1-32:leu1+ maf1-S59/60/61A-FLAG (maf1-3A) ura4-D18
S3D	Y5050	h- maf1::hphMX6 leu1-32:leu1+ maf1-S63E-FLAG ura4-D18
S3D	Y5040	h- maf1::hphMX6 leu1-32:leu1+ maf1-S63A-FLAG ura4-D18
S3D	Y5042	h- maf1::hphMX6 leu1-32:leu1+ maf1-S82/83/84E-FLAG (maf1-3E2) ura4-D18

S3D	Y4987	h- maf1::hphMX6 leu1-32:leu1+ maf1-S82/83/84A-FLAG (maf1-3A2) ura4-D18
S3D	Y5046	h- maf1::hphMX6 leu1-32:leu1+ maf1-S59/60/61/63/82/83/84E-FLAG (maf1-7E) ura4-D18
S3D	Y4965	h- maf1::hphMX6 leu1-32:leu1+ maf1-S59/60/61/63/82/83/84A-FLAG (maf1-7A) ura4-D18
S5A	Y2	h+ leu1-32 ura4-D18
S5A	Y3980	h+ maf1::hphMX6 leu1-32 ura4-D18
S5A	Y5218	h+ sfc3-1-12PK:kanMX6
S5A	Y5219	h+ sfc3-1-12PK:kanMX6 maf1::hhpMX6
S5A	Y5221	h+ sfc3-1-12PK:kanMX6 maf1::hhpMX6
S5B	Y2	h+ leu1-32 ura4-D18
S5B	Y3980	h+ maf1::hphMX6 leu1-32 ura4-D18
S5B	Y4208	h+ maf1-GFP:hphMX6 leu1-32 ura4-D18
S5B	Y4782	h+ tor2ts[L2048S]KanR leu1-32 ura4-D18
S5B	Y5062	h+ psk1::kanMX6 leu1-32 ura4-D18
S6	Y4627	h+ rad52-YFP:ura4+ leu1-32 ura4-D18
S6	Y4006	h+ maf1::hphMX6 rad52-YFP:ura4+ leu1-32 ura4-D18

Supplemental Table S2: Oligonucleotide primers used in this stuty

Figure	Name	Sequence
1A	tRNA(Ser+Met).f	TGT CCG AGT GGT TAA GGA GTT
1A	tRNA(Ser+Met).r	TGG GAC CTA CGG GTT ATG AG
1B	tRNA(Lys1).f	GGC TCA ATC GGT TTA GAG CG
1B	tRNA(Lys1).r	CTC GCA ACC TTC TGA TTA CCA T
1B	tRNA (Leu06).f	CAG TTG GCC GAG CGG TCT ATG
1B	tRNA (Leu06).r	TGA CCA GTG AGG GAT TCG AAC
3C,D,E	tRNA(Ser+Met).f	TGT CCG AGT GGT TAA GGA GTT
3C,D,E	tRNA(Ser+Met).r	TGG GAC CTA CGG GTT ATG AG
3C,D,E	tRNA (Leu06).f	CAG TTG GCC GAG CGG TCT ATG
3C,D,E	tRNA (Leu06).r	TGA CCA GTG AGG GAT TCG AAC
3C,D,E	tRNA (arg05).f	ACG CGT GGC GCA ATG GTA GC
3C,D,E	tRNA (arg05).r	CTT ACA CGA CGG GAC TCG AAC
6	tRNA (Leu05).f	GCT ATG CCC GAG TGG TCT AAG
6	tRNA (Leu05).r	TGC GGC CAG AGA GGT TCG AAC
6	tRNA (Leu06).f	CAG TTG GCC GAG CGG TCT ATG
6	tRNA (Leu06).r	TGA CCA GTG AGG GAT TCG AAC
6	tRNA (arg05).f	ACG CGT GGC GCA ATG GTA GC
6	tRNA (arg05).r	CTT ACA CGA CGG GAC TCG AAC
